# Organochlorine contamination enriches virus-encoded metabolism and pesticide degradation associated auxiliary genes in soil microbiomes

**DOI:** 10.1038/s41396-022-01188-w

**Published:** 2022-01-17

**Authors:** Xiaoxuan Zheng, Martin T. Jahn, Mingming Sun, Ville-Petri Friman, Jose Luis Balcazar, Jinfeng Wang, Yu Shi, Xin Gong, Feng Hu, Yong-Guan Zhu

**Affiliations:** 1 Soil Ecology Lab, College of Resources and Environmental Sciences, Nanjing Agricultural University, Nanjing, 210095 China; 2 Departments of Biochemistry, Zoology and Chemistry, University of Oxford, Oxford, OX1 3SZ United Kingdom; 3 Key Laboratory of Plant Immunity, Jiangsu Collaborative Innovation Center for Solid Organic Waste Resource Utilization & Jiangsu Key Laboratory for Solid Organic Waste Utilization, Nanjing, 210095 China; 4 University of York, Department of Biology, Wentworth Way, York, Y010 5DD United Kingdom; 5 Catalan Institute for Water Research (ICRA), Girona, 17003 Spain; 6 University of Girona, Girona, 17004 Spain; 7 Computational Genomics Lab, Beijing Institutes of Life Science, Chinese Academy of Sciences, 100101 Beijing, China; 8 State Key Laboratory of Soil and Sustainable Agriculture, Institute of Soil Science, Chinese Academy of Sciences, 71 East Beijing Road, Nanjing, 210008 China; 9 Research Center for Eco-environmental Sciences, Chinese Academy of Sciences, 18 Shuangqing Road, Haidian, 100085 Beijing, China

**Keywords:** Metagenomics, Soil microbiology, Microbial ecology

## Abstract

Viruses significantly influence local and global biogeochemical cycles and help bacteria to survive in different environments by encoding various auxiliary metabolic genes (AMGs) associated with energy acquisition, stress tolerance and degradation of xenobiotics. Here we studied whether bacterial (dsDNA) virus encoded AMGs are enriched in organochlorine pesticide (OCP) contaminated soil in China and if viral AMGs include genes linked to OCP biodegradation. Using metagenomics, we found that OCP-contaminated soils displayed a lower bacterial, but higher diversity of viruses that harbored a higher relative abundance of AMGs linked to pesticide degradation and metabolism. Furthermore, the diversity and relative abundance of AMGs significantly increased along with the severity of pesticide contamination, and several biodegradation genes were identified bioinformatically in viral metagenomes. Functional assays were conducted to experimentally demonstrate that virus-encoded L-2-haloacid dehalogenase gene (L-DEX) is responsible for the degradation of L-2-haloacid pesticide precursors, improving bacterial growth at sub-inhibitory pesticide concentrations. Taken together, these results demonstrate that virus-encoded AMGs are linked to bacterial metabolism and biodegradation, being more abundant and diverse in soils contaminated with pesticides. Moreover, our findings highlight the importance of virus-encoded accessory genes for bacterial ecology in stressful environments, providing a novel avenue for using viruses in the bioremediation of contaminated soils.

As the most abundant biological entities on earth, viruses of bacteria (bacteriophages referred as viruses from here on) play a critical role in modulating the ecology of microbial communities through lytic infection and lysogenic conversion of their bacterial hosts [1, 2]. Viruses significantly influence the biogeochemical cycles via the release of organic carbon and nutrients through host cell lysis, and in addition to core viral genes (i.e., genes encoding viral structural proteins [3]), they also encode various auxiliary metabolic genes (AMGs [4, 5]), which contribute the metabolic capacity and survival of their bacterial hosts. The role of AMGs has been especially well demonstrated with marine viruses that encode a diversity of AMGs involved in photosynthesis [6], translation machinery [7], carbon metabolism [8], phosphate metabolism [9] and sulfur cycle [10, 11]. Furthermore, sequencing of whole marine viral communities has revealed a clear involvement of viral AMGs in central carbon metabolism of host bacteria [10, 12]. Compared with the study of viral communities in marine ecosystem, the diversity and functional role of viral AMGs in soils are less well understood.

In soils, viruses reach abundances of up to ~10^9^ per gram of soil leading to frequent encounters with their host bacteria [13]. Similar to aquatic environments, viruses can regulate host bacterial densities, leading to indirect changes in the relative abundance of non-target bacterial taxa likely via release of niche space [14, 15]. Moreover, over longer time periods, viruses can coevolve with their host, following fluctuating selection dynamics [16] or patterns of local adaptation [17]. Viruses are also important mediators of horizontal gene transfer, promoting the transfer of antibiotic resistance genes, virulence factors and AMGs [18, 19]. However, these effects are less well understood at viral community level. Recent advances in viral purification have enabled a glimpse into soil viral communities of permafrost peatland [20, 21] and agricultural ecosystems [22, 23] based on metagenomics. These studies have demonstrated that viruses may alter the biogeochemical nutrient cycling [1, 2] and bacterial adaptation and evolution by carrying genes linked to carbon and nitrogen metabolism [20, 21]. Moreover, recent identification of atrazine chlorohydrolase *trzN* [24] and arsenic methyltransferase *arsM* [25] genes in soil-associated lysogenic viruses suggest that virus-encoded AMGs could shape bacterial metabolism under pollutant exposure. Therefore, we hypothesize, that contaminated soil microbiomes could contain a relatively higher abundance of viruses carrying AMGs linked to the degradation of pesticides and xenobiotics due to their potential benefit for the host bacteria.

Pesticide contamination imposes a serious threat to natural ecosystems and public health globally. China is the leading producer of organochlorine pesticides (OCPs), which are synthetic pesticides with vast applications in chemical and agricultural industries. OCPs are especially notorious due to their high toxicity, slow degradation and bioaccumulation [26]. Following the implementation of the Stockholm Convention, hundreds of pesticide plants in China were closed or re-located, and contaminated soils around the plants left untreated. As microbial communities are often capable of degrading OCPs, there is growing biotechnological interest to identify important genes and microbial taxa behind pesticide biodegradation. Heavy OCP contaminations have previously been shown to adversely impact soil bacterial diversity, composition, and activity [27, 28]. Prolonged exposure to contaminants has resulted in selection for bacteria that have evolved their own degradation enzymes, such as dehalogenases, which protect from the toxic effects of pesticides [29]. Interestingly, if also viruses can carry and encode such genes, pesticide exposure could create a strong positive selection for virus-encoded AMGs associated with pesticide degradation, potentially shifting soil microbiome community composition [30] by favoring bacterial and virus taxa that carry these genes.

To address this, we used a combination of metagenomics and direct experimentation to explore how pesticide exposure affects the abundance and type of bacterial and virus-encoded AMGs in the soil of former OCP production factory in Yangtze River Delta (China). We found that contaminated and clean control soils harbored very distinct bacterial and viral communities, and crucially, pesticide exposure was linked to higher diversity and abundance of virus-encoded metabolism and pesticide degradation AMGs. The functional activity of one candidate viral AMG, L-2-haloacid dehalogenase (L-DEX), was experimentally shown to improve bacterial growth at sub-inhibitory concentrations of haloacid, which is an important precursor of herbicides and insecticides. Together, our findings suggest that virus-encoded auxiliary genes could help bacteria to counteract pesticide stress, potentially explaining the benefits of virus carriage in stressful soil microbiomes.

## Results

### Characterization of the study site

Both bacterial and viral communities were recovered from three clean control (C1–C3) and six OCP-contaminated soil samples (S1–S6) from a formerly active OCP factory in the Yangtze River Delta (China) in the summer of 2018 (for experimental design and further info, see Supplementary Fig. 1 and Supplementary Table 1). Control samples were collected from nearby fallow fields outside the immediate factory area, and as no pesticides were detected, these soil samples are referred from here on as “clean” samples (Supplementary Table 2). The study site has a 30-year history of OCP production with the main contaminants being chlorobenzene, dichlorobenzene and nitrochlorobenzene. The factory was closed in 2007 and soils left untreated without bioremediation. According to the United States Environmental Protection Agency (EPA), the concentrations of toxic compounds within factory site were higher than the Screening Levels of residential soil or industrial soils (Supplementary Fig. 2a and Supplementary Table 2). As a result, the six OCP soil samples were categorized to “light” (S1–S3, total pesticide content varying form 281.3 ± 21.4 to 509.8 ± 28.7 mg kg^−1^), and “heavy” (S4–S6, total pesticide content varying from 1083.7 ± 40.4 to 4595.8 ± 344.0 mg kg^−1^) contaminated samples.

### Overview of bacterial communities in clean and OCP-contaminated soils

**Fig. 1:**
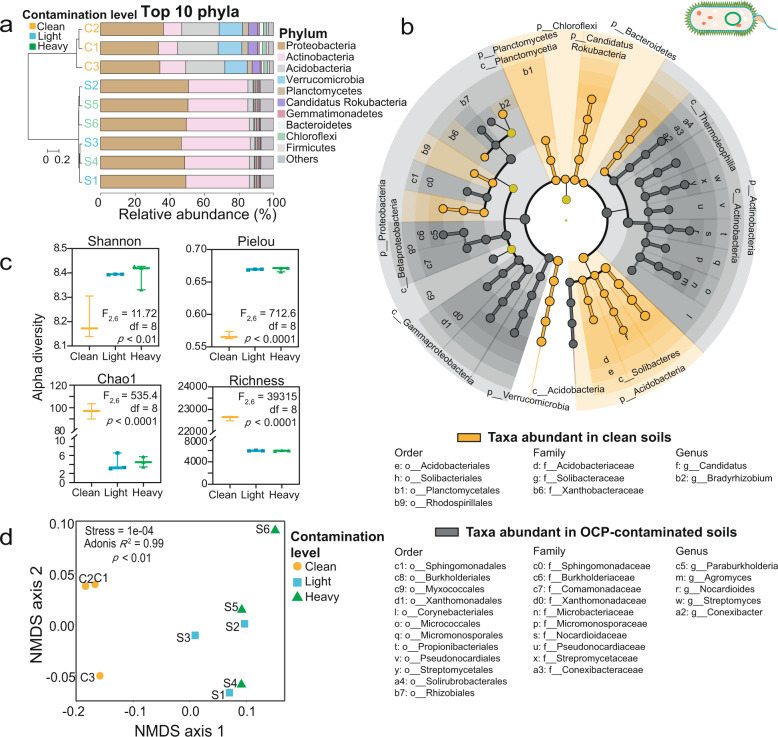
Differences in bacterial communities in clean and OCP-contaminated soils. **a** Relative abundance of the top 10 abundant bacteria phyla in clean (C1–C3) and OCP-contaminated (S1–S6) soils. The left *Y*-axis shows UPGMA clustering based on Bray–Curtis distances. **b** Linear discriminant analysis comparing bacterial abundance differences between clean and OCP-contaminated soils at phyla to genera levels (from outer to inner circles; LDA score threshold > 3.8; only clearly classified taxa shown). Orange and gray colors represent the taxa abundances that were significantly different in clean versus OCP-contaminated soils, respectively (phylum and class taxa information is displayed in the legend below the cladogram). **c** Differences in alpha diversity between clean (C1–C3), and OCP-contaminated soils (Light contamination: S1–S3; Heavy contamination: S4–S6). **d** NMDS analysis comparing differences in community composition between clean (C1–C3) and OCP-contaminated soils (Light contamination: S1–S3; Heavy contamination: S4–S6). ANOVA followed by Tukey’s multiple comparisons test was used to compare difference between groups.

In order to characterize the impact of OCP contamination on soil bacterial communities, we investigated the relative abundance of different bacterial taxa between clean and OCP-contaminated soil microbiomes (Supplementary Table 3). We identified 29,902 and 8,371 bacterial taxa in clean and OCP-contaminated soils, respectively and the bacterial rarefaction curve slope was similar (Supplementary Fig. 2b and Supplementary Table 3- “Taxonomy”). Clean soils were dominated by Proteobacteria (34.7%), Acidobacteria (22.5%), Verrucomicrobia (13.4%), and Actinobacteria (12.2%), accounting for 82.8% of the total bacterial diversity. In contrast, while the relative abundances of Proteobacteria (49.2%) and Actinobacteria (36.5%) increased in OCP-contaminated soils, the abundances of Acidobacteria (2.8%) and Verrucomicrobia (0.5%) clearly decreased (all phyla together representing 88.5% of the total bacterial diversity, Fig. 1a). Notably, three times more bacterial taxa were enriched in OCP-contaminated soils (gray dots in Fig. 1b, 27 bacterial taxa) as compared to those that showed decreased relative abundances (orange dots in Fig. 1b, 9 bacterial taxa). Positively affected taxa included *Paraburkholderia, Streptomyces* and *Nocardiodes* genera (Fig. 1b) and negatively affected *Candidatus* and *Bradyrhizobium* genera (LDA Score > 3.8; Fig. 1a, b). OCP exposure was also associated with a reduction in the total number of bacterial taxa, and lowered community richness (i.e., lower ACE, Chao1, Richness) but higher community evenness (i.e., higher Shannon, Simpson and Pielou indexes; Fig. 1c and Supplementary Fig. 2c). As a result, OCP exposure clearly changed bacterial community composition in soil microbiomes (NMDS analysis: Adonis *R*^*2*^ = 0.99, *p* < 0.05; Fig. 1d), while no difference was observed between light and heavy contaminated soils (Stress value = 1e−04 < 0.05; Fig. 1d; also verified with UPGMA analysis, Fig. 1a, c, d).

### Overview of viral communities in clean and OCP-contaminated soils

**Fig. 2:**
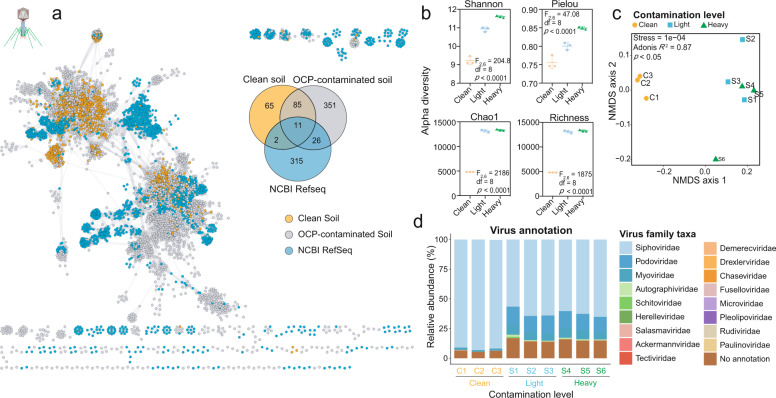
Differences in viral communities in clean and OCP-contaminated soils. **a** A gene-sharing network for viral contigs (>10 kb) isolated from clean (orange) and OCP-contaminated soils (gray) and NCBI RefSeq database (blue). Nodes (circles) represent viral genome contigs and edges indicate shared protein content. The Venn diagram on the top right corner of (**a**) shows shared and unique viral clusters (VCs) resulting from vConTACT 2.0 between clean, OCP-contaminated and RefSeq sequences. **b** Differences in alpha diversity between clean (C1–C3), and OCP-contaminated soils (Light contamination: S1–S3; Heavy contamination: S4–S6). **c** NMDS analysis between clean (C1–C3), and OCP-contaminated soils (Light contamination: S1–S3; Heavy contamination: S4–S6). **d** Bar plot showing viral taxonomic composition at the family level in clean (C1–C3), and OCP-contaminated soils (Light contamination: S1–S3; Heavy contamination: S4–S6). ANOVA followed by Tukey’s multiple comparisons test was used to compare difference between groups.

Based on transmission electron microscopy (TEM), tailed and non-tailed viruses were the main virus types detected in all soil samples (Supplementary Fig. 3a). To assess the diversity and functioning of viral communities, a total of 19,292 viral contigs (>1 kb) were obtained using metagenomic sequencing (Supplementary Table 4- “Contigs”). A higher number of viral contigs was recovered from OCP-contaminated (*n* = 13,905) compared to clean soils (*n* = 5,387). Contigs clustered into 18,458 vOTUs and rarefaction analysis showed that the discovered viral diversity saturated in both clean and contaminated samples, which indicates that our sequencing depth was adequate for capturing most common viruses in both samples (Supplementary Fig. 3b). vOTUs representing long sequences of more than 10k bp (*n* = 4,572) were further compared to viral NCBI RefSeq v85 genomes. This approach allowed to identify 909 viral clusters (VCs) with approximate genus level classifications (Fig. 2a; Supplementary Table 4- “Virus taxonomy” and “Network_data_1”). Clean soil viral communities (Network Density = 0.021) had a more compact network structure than NCBI RefSeq genomes (Network Density = 0.016), while OCP-contaminated soil viral communities had relatively more dispersed networks (Network Density = 0.006). However, both clean (Clustering coefficient = 0.637; Avg. number of neighbors = 23.379) and contaminated (Clustering coefficient = 0.632; Avg. number of neighbors = 17.311) soil viral communities had lower clustering coefficients and average number of neighbors than NCBI RefSeq database network (Clustering coefficient = 0.815; Avg. number of neighbors = 35.502; Supplementary Table 4- “Network_data_1_parameters”). Furthermore, viral communities from the clean and OCP-contaminated soils and NCBI RefSeq database clearly fell into in 163,473 and 354 VCs, respectively. Notably, our viral samples shared only 39 VCs with the NCBI RefSeq database, indicating that currently culturable viruses cover only a small fraction of the contaminated soil viruses (Fig. 2a). While 96 VCs were shared between clean and OCP-contaminated viral communities, 351 VCs were exclusively detected only in OCP-contaminated soils (Fig. 2a). Overall, OCP-contaminated soil viral communities were more diverse (i.e., higher Chao1 and Richness indexes), and more even (i.e., higher Shannon, Simpson and Pielou indexes; Fig. 2b and Supplementary Fig. 3c). Similar to bacterial communities, clean and OCP-contaminated soil viral communities had distinct community structures, while no differences between light and heavy contamination levels was found (Fig. 2c and Supplementary Fig. 3d). Although the majority of viruses could be assigned to known viral families using vConTACT 2.0 classification and majority-rules approach, 14% of these were novel viruses (Supplementary Fig. 3e and for details see Methods). Specifically, the number of novel vOTUs was higher in contaminated (16.1%; 2,197 of 13,656 sequences) compared to clean soils (8.7%; 421 of 4,842 sequences). The relative abundance of unannotated viruses was also higher in OCP-contaminated (14.4% in average) compared to clean soils (6.0% in average). *Siphoviridae* was the most dominant family in both clean (92.0% in average) and OCP-contaminated (62.2% in average) soils, while *Podoviridae* (F_2,6_ = 269.2, *p* < 0.0001) and *Myoviridae* (F_2,6_ = 48.8, *p* = 0.0002) had higher relative abundances in OCP-contaminated soils irrespective of the contamination level. Notably, five viral families (*Schitoviridae, Demerecviridae, Chaseviridae, Fuselloviridae*, and *Pleolipoviridae*) were exclusive to OCP-contaminated soils, while three viral families (*Microviridae, Rudiviridae*, and *Paulinoviridae*) were only found in clean soils, respectively (Fig. 2d). Together, these results suggest that OCP-contaminated soils were associated with distinct bacterial and viral communities.

### OCP-contaminated soils had a higher number of broad host range viruses

**Fig. 3:**
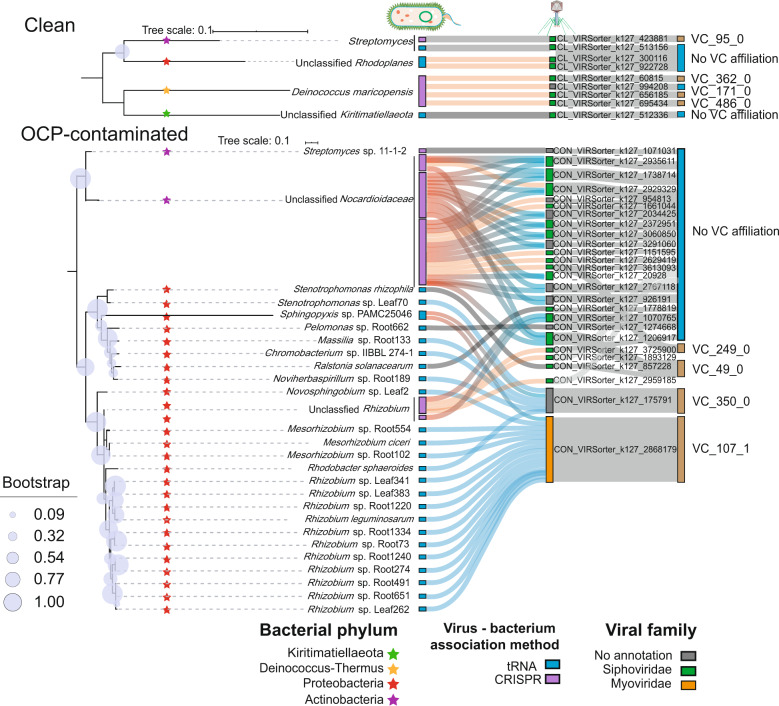
Predicted virus-host associations based on tRNA matches (blue) and CRISPR spacer linkages (purple) in clean and OCP-contaminated soils. Left: bacterial phylogenetic tree based on 16S rRNA gene sequences where differently colored stars denote for different bacterial phyla. Boxes after bacterial taxa denote for method for identifying virus-host association (tRNA and CRISPR). Right: viral contigs (at family level) originating from different viral clusters (blue VC groups denote for viruses that did not have no viral cluster affiliation). Gray, red and blue connecting lines show associations defined as specialist (one bacterial host and one virus), generalist (one bacterial host and multiple viruses) and polyvalent (multiple bacterial hosts and one virus) virus-bacteria associations, respectively.

To investigate potential associations between viruses and bacteria, we pooled light and heavy OCP-contaminated soil viral communities and compared them with the clean soil samples. Based on the tRNA matches and clustered regularly interspaced short palindromic repeats (CRISPR) spacer linkages (see Methods), we could link 30 bacterial host taxa to their respective viruses in contaminated soils, in contrast to 4 host taxa links observed in clean soils (Fig. 3). More specifically, *Streptomyces*, *Rhodoplanes* and *Deinococcus maricopensis* bacteria in clean soils, and *Nocardioidaceae*, *Rhizobiaceae* in *Sphingopyxis* sp. PAMC25046 bacteria in OCP-contaminated soils, were associated with different viral contigs from various VCs (Fig. 3). Interestingly, viruses with broad host ranges (viral contigs associated with multiple host taxa) were only detected in OCP-contaminated soils: 14 out of the total 26 viral contigs observed in OCP-contaminated soils were associated with a total of 23 bacterial taxa, mainly including *Nocardioidaceae* and *Rhizobium*. Among these viral contigs, *Siphoviridae* accounted for a large proportion (25/35). Moreover, two viral contigs (CON_VIRSorter_k127_175791 and CON_VIRSorter_k127_2868179) showed generalism (potential polyvalent phages), being associated with several host bacterial families (Fig. 3 and Supplementary Table 5). Additional information on host-virus associations was derived by querying matching viral sequences in JGI public database. Overall, we were able to link 10,932 bacterial host records representing 19 bacterial phyla with 4,041 viral contigs and the relative abundance of predicted bacterial hosts was positively correlated with their relative abundance (Supplementary Fig. 4). However, no potentially new virus-host links were found based on comparison with previously reported literature [20] or NCBI Genbank and JGI Viral Sequence databases. Together these results suggest that viruses were associated with a higher number of bacterial hosts in OCP-contaminated compared to clean soils.

### Virus-encoded auxiliary genes are involved in both metabolism and pesticide degradation

**Fig. 4:**
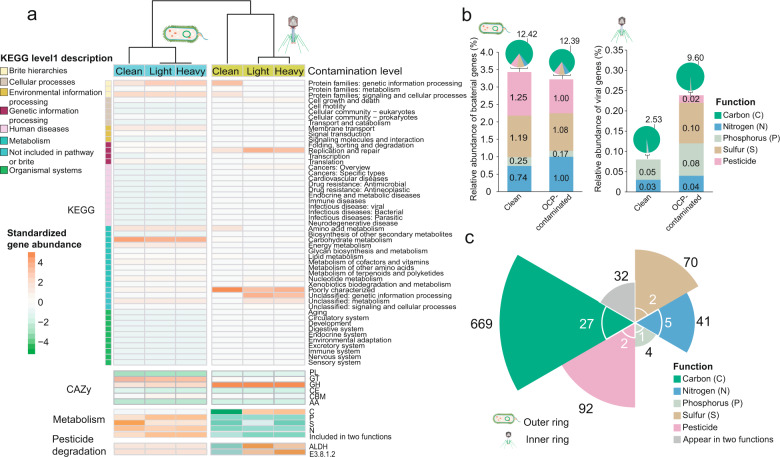
Functional annotation and relative abundances of bacterial and virus-encoded genes in clean and OCP-contaminated soils. **a** Heatmap shows the relative abundance of functional genes annotated by KEGG and CAZy databases, and known genes linked to nutrient metabolism and pesticide degradation in bacterial and viral metagenomes in clean (C1–C3), and OCP-contaminated soils (Light contamination: S1–S3; Heavy contamination: S4–S6). **b** Differences in relative functional gene abundances in clean and OCP-contaminated soils based on bacterial and viral metagenomes. Genes linked to carbon metabolism (green) are only shown in pie charts, while bar charts how relative abundances of relatively less abundant metabolism and pesticide degradation genes. **c** Number of functional genes associated with bacteria (outer ring; black text) and viruses (inner ring; white text). Genes linked to carbon (C), nitrogen (N), phosphorus (P) and sulfur (S) metabolism and pesticide-degradation are shown in green, blue, light blue, brown and pink colors, respectively. Gray color denote genes that were associated with two any functions.

To explore the contribution of viruses for the ecology of bacterial communities, we compared the functional annotations of both bacterial and viral sequences in clean and OCP-contaminated soils. Based on the KEGG database annotations [31], the abundance distribution of bacterial functional genes was similar regardless of whether they originated from clean or OCP-contaminated soils (Fig. 4a, Supplementary Table 3- “KEGG annotation of bacteria” and Supplementary Table 6), and were mainly annotated under “Metabolism” and “Genetic and environmental information processing” categories (Fig. 4a). Most of the viral genes (about 50% of predicted open reading frames (ORFs)) could only be annotated as “unclassified and poorly characterized” using KEGG database, while other genes (about 17.5% of ORFs) belonged mainly under “Replication and repair”, “Cell growth and death”, and “Human disease” categories (Supplementary Table 7- “KEGG annotation of virus”). Annotated viral genes were often predicted to be involved in bacterial metabolism (Supplementary Fig. 5a). To investigate this in more detail, we compared bacteria- and virus-encoded carbohydrate-active enzymes using a CAZy database [32] (Supplementary Table 3-“CAZy annotation of bacteria” and Table 7- “CAZy annotation of virus”). Overall, the number of annotated viral and bacterial CAZy subfamilies was higher in OCP-contaminated soils (Wilcoxon rank sum test; viruses: *p* = 0.01, bacteria: *p* = 0.03), with glycoside hydrolases (GH), glycosyl transferases (GT) and carbohydrate-binding modules (CBM) being the most often annotated functional groups (Supplementary Fig. 5b, c). To assess the role of viruses for bacterial metabolism, viral genes involved in nutrient transformation and pesticide degradation were selected and fitted into relevant metabolic pathways in soil bacterial communities [33] (Supplementary Table 7- “Summary of selected genes”). While the diversity (F_2,9_ = 6.427e−005, *p* > 0.05) and relative abundance (F_2,9_ = 8.811e-006, *p* > 0.05) of genes linked to metabolism (carbon (C), nitrogen (N), phosphorus (P) and sulfur (S)) did not differ between clean and OCP-contaminated soils (Fig. 4b), virus-encoded metabolic genes were more diverse and abundant in OCP-contaminated (35 gene categories) compared to clean soils (22 gene categories) (Fig. 4b and Supplementary Table 6). Specifically, denitrifying *norD* and *norQ* genes [34], and hydrogen sulfide metabolism-related, *cysD, cysH* genes [10] (which also have been found in human and environmental systems recently [35]), were only detected in viruses exposed to OCP-contamination but not in clean soils. Similarly, the number and relative abundance of carbon-cycle associated genes were found in higher numbers in the viral genomes of OCP-contaminated soils (OCP-contaminated soils: *n* = 27 with a total relative abundance of 9.60%; clean soils: *n* = 19 with a total relative abundance of 2.53%; Fisher’s exact test, *p* = 0.0001, Fig. 4b). Of the 136 bacterial genes linked to pesticide degradation, two gene categories were exclusively encoded by viruses found in OCP-contaminated soils. These included aldehyde dehydrogenase (ALDH) and L-2-haloacid dehalogenase (EC:3.8.1.2), which are responsible for the transformation of chlorobenzene and chloroalkene, respectively (see the next result section). Interestingly, relative abundances of pesticide degradation genes were positively correlated with carbon metabolism genes in both viral and bacterial communities (Pearson|*r*| > 0.6 and *p* < 0.05) consistent with CAZy signatures (Supplementary Fig. 6a, b). Even though a relatively small number of functional genes were shared between bacteria and viruses (Fig. 4c), virus-encoded genes covered a range of bacterial metabolic activities and pesticide degradation pathways. We thus compared the changes in viral AMGs and core functional genes (genes linked to viral replication and viral structure) between clean and OCP-contaminated soils (see Methods for details, Supplementary Table 8). The mean AMG abundances were significantly greater with viruses compared to bacteria (Paired t-test, *p* < 0.001; Supplementary Fig. 6c) irrespective of the level of pesticide contamination (F_2,210_ = 0.018, *p* > 0.05; Supplementary Fig. 6d). Moreover, the diversity of viral AMGs was higher in OCP-contaminated compared to clean soils (ANOVA followed by Tukey’s multiple comparisons test, F_2,6_ = 14.52, *p* = 0.005), and the abundance of viral AMGs was significantly higher in heavy compared to light OCP-contaminated soils (ANOVA followed by Tukey’s multiple comparisons test, F_2,6_ = 4.93, *p* < 0.05; Supplementary Fig. 7a). The AMG differences were more pronounced at the contig versus VC level (Supplementary Fig. 7b), indicating that AMG transfer may be more frequent between viruses that are phylogenetically closely related. Together, our findings demonstrate that genes linked to both bacterial metabolism and pesticide degradation were enriched in viral metagenomes in OCP-contaminated soils.

### Functional validation of virus-encoded genes in pesticide degradation

**Fig. 5:**
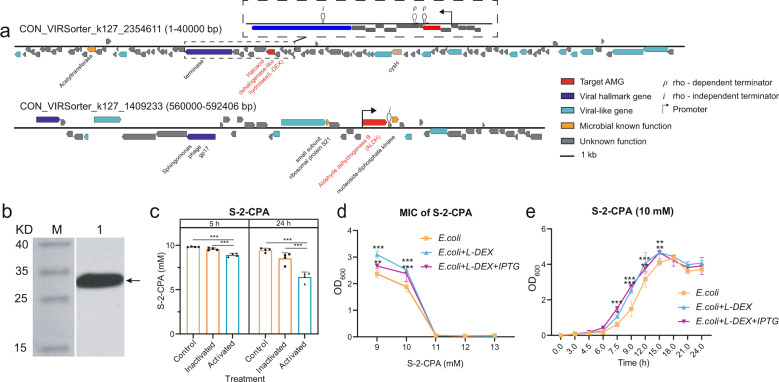
Functional validation of virus-encoded pesticide degradation gene. **a** Genome maps of viral contigs showing the location of L-DEX (top) and ALDH (bottom) genes on red. Viral hallmark genes, viral-like genes, microbial known function genes and hypothetical but unknown function genes are highlighted in deep blue, light blue, yellow and gray, respectively. **b** Western plot analysis showing the L-DEX products expressed in *E. coli*. Lanes show marker (M) and product (1). **c** Enzymatic activity (S-2-CPA breakdown) of purified L-DEX in control (no protein added), inactivated (protein deactivated by high temperature) and activated (protein added) treatments, respectively. **d** Minimum inhibitory concentration (MIC) of S-2-CPA for *E. coli*. **e** *E. coli* growth curve in 10 mM S-2-CPA in LB. In **d** and **e**, *E.coli, E.coli* + *L-DEX* and *E.coli* + *L-DEX* + *IPTG* show the donor strain *E. coli* ArcticExpress without L-DEX gene, *E. coli* ArcticExpress with L-DEX gene and *E. coli* ArcticExpress with L-DEX gene induced by 0.4 mM IPTG, respectively. ANOVA followed by Tukey’s multiple comparisons test was used to compare difference between groups.

The presence of two genes encoding aldehyde dehydrogenase (ALDH) and L-2-haloacid dehalogenase (L-DEX, EC:3.8.1.2) in viral genomes was confirmed using a more refined analysis (Supplementary Fig. 8a and Supplementary Table 9). Specifically, CheckV (v 0.8.1) [36] and VIBRANT (v 1.2.0) [37] were used to check the integrity of the viral genome and location of these genes regarding nearby viral genes. Both genes were flanked by viral hallmark genes on both sides (VIRSorter category 2; genes linked viral replication and structure that could be identified with high confidence), indicating a strong evidence for viral origin (Fig. 5a and Supplementary Table 9-“Gene information”). Aldehyde dehydrogenase (ALDH) encoded by CON_VIRSorter_k127_1409233 was assigned by DRAM-v (v 1.2.0) [38] as “MK”, which suggests it is a known auxiliary metabolic gene. However, DRAM-v did not recognize L-2-haloacid dehalogenase (L-DEX) as an AMG even though this gene was given an auxiliary score of 2, which means that dehalogenase degradation does not belong to usual metabolic processes. One reason for this might be that it has not received much attention in previous studies and is missing from DRAM-v database (Supplementary Table 9-“DRAM-v information”). A promoter (*p* = 0.0005) and a Rho-independent terminator (score = −14.3) were located around ALDH. Similar with L-DEX, a promoter (*p* = 0.001) and a Rho-independent terminator (score = −13.5) were found upstream and downstream of this gene. Moreover, we found two potential Rho-dependent terminators within the L-DEX gene that could have affected the transcription of this or other potentially overlapping viral genes (Supplementary Table 9 – “Gene information”). Together, our findings suggest that the identified ALDH and L-DEX genes were of viral origin and likely under positive selection as they were only found in contaminated soils.

L-2-Haloacid dehalogenase (L-DEX) belongs to the haloacid dehalogenase-like (HAD) hydrolases, and catalyzes the hydrolytic dehalogenation of L-2-haloacids, which is an important precursor for the synthesis of pesticides, including Hexachlorocyclohexane (HCH) and D-2-hydroxyacids [39]. In addition, one of its substrates, 2-chloropropionic acid, is also a commonly used broad-spectrum herbicide. Phylogenetic analysis showed that the virus-encoded protein was evolutionarily distinct from the most bacterial HAD reference sequences, and shared the closest similarity with *Mycolicibacterium* (41.86% protein similarity, 48% coverage) recovered from S1 sample in our metagenomic dataset. *Mycolicibacterium* has previously been reported as a typical pesticide degradation genus [40] and had an average abundance of 0.93% in OCP-contaminated soils (Supplementary Fig. 8b, Supplementary Table 9- “L-DEX BLASTp query”; a much higher relative to clean soils with 0.30% relative abundance, Supplementary Table 3- “Taxonomy”). We found that the L-2-haloacid dehalogenase encoded by the virus comprised all the active sites (12 out of 12) of the HAD_L2-DEX conserved domain model (cd02588), and its catalytic core residues were highly conserved across the entire HAD phosphatase family, which aggregates into four signature motifs (Supplementary Fig. 8c). The first Asp of motif I is the essential Asp (D) nucleophile, and a conserved Ser (S) of motif II helps to orient the substrate for nucleophilic attack by forming a hydrogen bond with its transferring phosphoryl group. Motif III takes a conserved Lys (K) residue as core structure, which stabilizes the negative charge of the reaction intermediate together with Thr(T) of motif I. Together with the Asp (D) residues of motif I, the motif IV, acidic Asp (D) residues are involved in the coordination of Mg^2+^ (Supplementary Fig. 8c). In addition, the structural model prediction of virus-encoded L-2-haloacid dehalogenase at Phyre2 [41] showed 100% confidence (Supplementary Fig. 8a and Supplementary Table 9). The identified virus-encoded L-2-haloacid dehalogenase may thus represent a novel halogenic acid dehalogenase.

The activity of virus-encoded L-2-haloacid dehalogenase (L-DEX) was further validated experimentally. The synthesized gene L-DEX was cloned into pET-32a (+) plasmid, and chemically transformed into the acceptor *E. coli* for expression. Upon activation of virus-encoded L-DEX in *E. coli*, we were able to detect a 30.9-kDa protein (including a 12.6-kDa protein with N-6*His tag) and 18.3-kDa protein in western plot analysis (Fig. 5b). The degradation activity of the purified protein was investigated experimentally by testing if the virus-encoded L-DEX could break down two haloacid precursors, monochloroacetate (MCA) and S(L)-2-chloropropionic acid (S-2-CPA), leading to detoxification of the environment. In support for this, L-DEX expressed in *E. coli* cells could reduce the concentrations of MCA and S-2-CPA by 13.8% and 11.0% after 5-h incubation, respectively (ANOVA followed by Tukey’s multiple comparisons test, MCA: F_2,9_ = 44.23, *p* < 0.0001; S-2-CPA: F_2,9_ = 44.53, *p* < 0.0001). Further, MCA and S-2-CPA concentrations showed 60.0% and 37.8% decline after 24-hour incubation with the protein, respectively (ANOVA followed by Tukey’s multiple comparisons test, F_2,9_ = 150.30, *p* < 0.0001; S-2-CPA: F_2,9_ = 37.97, *p* < 0.0001; Fig. 5c and Supplementary Fig. 9a). Moreover, even though the presence of L-DEX plasmid did not change the minimum inhibitory concentration (MIC) of *E. coli* (8 mM MCA and 11 mM S-2-CPA) (Fig. 5d and Supplementary Fig. 9b), it allowed improved growth at subinhibitory S-2-CPA concentrations (ANOVA followed by Tukey’s multiple comparisons test, F_8,30_ = 9.49, *p* < 0.0001). Moreover, the expression of virus-encoded L-DEX in *E. coli*  allowed bacterium to enter the exponential phase faster than without the plasmid at sub-MIC 10 mM S-2-CPA concentration (ANOVA followed by Tukey’s multiple comparisons test; F_20,66_ = 4.07, *p* < 0.0001, Fig. 5e; F_20,66_ = 2.43, *p* = 0.0037, Supplementary Fig. 9c). Together, these result show that viral-encoded L-2-haloacid dehalogenase formed an active protein that was beneficial for bacteria by breaking down pesticides and improving the growth of L-DEX plasmid containing *E. coli* cells.

## Discussion

While bacterial and virus metagenomes have been extensively studied in aquatic systems [42, 43] and in the human gut [44, 45], soil ecosystems remain less well explored. We employed metagenomic sequencing of soil microbiomes [46] to demonstrate clear associations between pesticide contamination and bacterial and viral community diversity, composition and functioning. Specifically, we found that OCP-contaminated soils had distinct bacterial communities, including a higher relative abundance of taxa previously linked to pesticide degradation, such as *Paraburkholderia*, which have been found to degrade single- and multi-ring aromatic hydrocarbons [47], and *Streptomyces* and *Nocardiodes* that are considered the most representative genera of organic pesticide degrading bacteria [48]. Moreover, the viral communities of OCP-contaminated soils were more diverse, contained a high number of unique viral taxa and had a higher number of predicted host bacterial taxa associations, which could be indicative of relatively stronger virus-bacteria co-dependencies. In support for this, bacterial genes linked to metabolism and pesticide degradation were enriched in OCP-contaminated soil viral metagenomes, while no effect on these gene abundances were observed in bacterial metagenomes. Previous studies conducted in marine [12, 49, 50] and soil [21, 51, 52] ecosystems, have identified a variety of auxiliary metabolic genes in bacterial and virus metagenomes based on in Pfam, KEGG, and CAZy databases. Here we show that pesticide degradation could be one driver enriching virus-encoded AMGs in soil microbiomes. Likely explanation for this is that these viral AMGs are likely to be beneficial for bacteria by alleviating the toxic effect of pesticides [53] or by helping to acquire energy through pesticide degradation [54]. For example, high CAZyme abundances observed in OCP-contaminated soils suggest that viruses could regulate carbon cycling in addition to lysing host cells (i.e., “viral shunt”) [13, 52, 55, 56], potentially affecting bacterial nitrogen, phosphorus, and sulfur metabolism [1] and pesticide degradation via microbial (co)metabolism [57]. To further test the potential role of virus-encoded AMGs for pesticide degradation, we bioinformatically identified and cloned virus-encoded L-2-haloacid dehalogenase to *E. coli*. The purified proteins were active at degrading monochloroacetate (MCA) and S(L)-2-chloropropionic acid (S(L)-2-CPA) haloacid precursors. Furthermore, L-DEX plasmid carriage improved *E. coli* growth at sub-MIC pesticide concentrations. Together, this functional validation suggests that virus-encoded auxiliary genes that help bacteria to survive under pesticide stress. In the future, it would be interesting to compare several contaminated and clean soils to explore if identified phage-encoded functions are unique to industrial sites or if these genes can also be discovered in natural soils. This would help to address if these functions have evolved only recently due to human pesticide manufacturing or if they have more distant evolutionary origin. Furthermore, experimental evolution approaches could be used to directly test if pesticide exposure could shift bacteria-phage interactions along with the parasitism-mutualism continuum, turning antagonistic viruses to beneficial “endosymbionts” via provision of fitness benefits in stressful environments.

In conclusion, our results highlight the importance of viral communities for the bacterial ecology in soil microbiomes. Moreover, we show that viruses could provide a novel tool for bioremediation of contaminated soils. As organochlorine pesticides are notoriously highly toxic, slow at degrading and often accumulate in food chains, identifying functional biodegradation genes and associated microbial taxa has a great biotechnological interest. We suggest that viruses could provide a novel tool for bioremediation of contaminated soils by providing important AMGs for their host bacteria.

## Methods

### Site description and sample collection

Bulk soil samples were collected nearby a closed pesticide factory located at Jiangsu province, China (N’120.228193, E’31.758075). The soil had been subjected to continuous exposure of organochloride pesticides (OCPs) between 1975 and 2007 due to extensive pesticide production and lack of sewage treatment facilities. The site was left in natural state and recovery after the closure of the factory in 2007. According to the preliminary background investigation, the overall area of the site is ~169,620 m^2^, with 40,708 m^2^ area categorized as OCP-contaminated are according to the national soil environmental quality risk control standard (GB15618-2018). Due to the high annual OCP production (more than 20,000 tons in 2006), the site is mainly contaminated by chlorobenzene, dichlorobenzene and nitrochlorobenzene with concentrations ranging from 281.3 ± 21.4 to 4595.8 ± 344.0 mg kg^−1^. The factory is located at the Yangtze River Delta, which has the highest number of pesticide production plants nationally, and thus well represents a typical Chinese chemical plant that were operational during the past decades. Six soil samples were collected from areas with varying pesticide contents in the soil (S1–S6). Based on the preliminary site investigation in 2019 (Supplementary Fig. 1), three clean control soil samples (C1–C3) without pesticide exposure were collected from the nearby fallow land, which located ~1.5 km away from the former pesticide factory. At each sampling area, 2 kg soil was collected randomly from five aliquots at the depth of 0-20 cm with three composite replicates. Soil samples were stored in sterile 1-L polypropylene Falcon tubes at 4 °C and transported to the laboratory before storage at −80 °C prior to analysis. While storage at −80 °C may have increased virus mortality, this effect was the same for all the samples and did not create systematic bias to our results.

### Determining soil physicochemical properties and pesticide contents

Soil samples were grounded through 2-mm sieve and analyzed for soil physico-chemical properties [58], including soil Ph, cation exchange capacity (CEC), soil organic matter (SOM), total nitrogen (TN), total phosphorus (TP) and available sulfur (Supplementary Table 2). The pesticide contents were determined according to methods described by Sun et al. [59] and Ye et al. [60]. Briefly, pesticide determination procedures were carried out with an accelerated solvent extractor system (ASE-200; Dionex, USA) by extracting into dichloromethane, followed by GC-MS analysis (Agilent GCMS 6890N-5973 N, USA).

### Bacterial metagenomic sequencing and analysis

FastDNA Spin kit for soil (MP Bio) was used to extract the total DNA from all soil samples following manufacturer’s instructions. Extracted DNA samples were sent to Shanghai Personal Biotechnoloy Co., Ltd. (Shanghai, China) for high throughput sequencing. Nine libraries of 400 bp insert-size fragments were constructed for whole-genome shotgun approach, and paired-end (PE, 2 × 150 bp) sequencing was carried out on a HiSeq X platform (Illumina, San Diego, CA, USA). After quality screening conducted by Cutadapt (v1.2.1) [61], a total of ~8.8 billion clean reads (~0.8 billion per clean soil samples and ~1.06 billion per pesticide-contaminated soil samples) were obtained and used for de novo assembly by Megahit (v 1.2.6) (https://hku-bal.github.io/megabox/) [62] with k-mer~ parameter setting [27, 127] (Supplementary Table 1). Open reading frames (ORFs) were predicted using MetaGeneAnnotator [63], followed by redundancy elimination by using CD-HIT (v 4.8.1) [64] based on 90% sequence similarity and 90% coverage. High quality reads were mapped to the contigs using BWA (v 0.7.17, BWA-MEM algorithm) [65] with default parameters, and the obtained contig abundance and gene TPM (Transcripts Per Kilobase of exon model per Million mapped reads) values were calculated by Soap.coverage (v 2.7.9, http://soap.genomics.org.cn/) and a custom script (https://github.com/EnvGen/metagenomics-workshop/blob/master/in-house/tpm_table.py), respectively. Further bacterial taxonomy annotation was carried out using the lowest common ancestor (LCA) algorithm in MEGAN 5 [66] based on NCBI-NT reference database via BLASTn (Nucleotide collection, ftp://ftp.ncbi.nih.gov/blast/db/, v2016-6-19, E-value ≤ 10^−5^; Supplementary Table 3). Bacterial 16S rRNA gene sequences were downloaded from Silva (https://www.arb-silva.de/) and NCBI database to construct phylogenetic in MEGA 7 [67], which was visualized in iTOL [68]. Bacterial gene function annotations are described later in the methods along with viral gene function annotations.

### Virus DNA extraction and sequencing

Virus DNA was extracted following methods described by Trubl et al. [69] and Adriaenssens et al. [70] with following modifications. Briefly, sub-soil samples (300 g) were homogenized through 0.25 mm-sieve, and mixed with 1 liter 1% (*w/w*) of potassium citrate buffer (10 g L^−1^ C_6_H_5_K_3_O_7_, 1.92 g L^−1^ Na_2_HPO_4_·12H_2_O and 0.24 g L^−1^ KH_2_PO_4_; pH = 7). The mixture was first incubated at 4 °C for 15 min, then sonicated (100 W, 47 kHz) on ice for 3 min with 30 s of manual shaking at every minute. After first centrifugation (7,000 rpm, 10 min), the supernatant was transferred to another tube and centrifuged again at 7000 rpm for 15 min. The yielded supernatant was filtered sequentially through 0.45-μm and 0.22-μm filters (Anpel hydrophilic PTFE syringe filter, China) to remove remaining non-virus like particles. The extract was enriched by using tangential flow filtration technology (TFF, Sartorius Vivaflow50 30,000 MWCO PES, USA). Virus samples were examined for purity and morphology under transmission electron microscope by 1% uranyl acetate staining (FEI Tecnai G2 Spirit Bio TWIN, USA) (Supplementary Fig. 1). The virus DNA extracts were treated with DNase I [TaKaRa Recombinant DNase I (RNase-free) 2270A]: RNase A (Takara Ribonuclease A 2158) mixture in 2:1 ratio at 37 °C for 30 min to remove non-encapsulated DNA fragments. The presence of bacterial DNA was examined by 16S rRNA gene PCR. The solution was then used for virus DNA extraction using Takara MiniBEST Viral RNA/DNA Extraction Kit Ver.5.0, and viral DNA concentrations were determined using Qubit 3.0 fluorometer (Invitrogen, Waltham, Massachusetts).

The extracted virus DNA was subjected to whole-genome amplification (KAPA HiFi HotStart ReadyMix) to meet the metagenome sequencing requirements. It should be noted that this method could have introduced unavoidable but small sequencing bias. The nine amplification products were sent for metagenomic sequencing, and each library of 400 bp insert-size fragments yielded 150 bp paired-end reads using a HiSeq 4000 platform (Illumina, San Diego, CA, USA).

### Virus identification

After quality control with Cutadapt (v 1.2.1), a total of ~9.6 billion clean reads (~1.06 billion per sample) were used for de novo co-assembly of viral sequences [20, 21, 71] to address potential virome between clean and OCP-contaminated viromes using Megahit with k-mer ~ parameter setting [27, 127] (Supplementary Table 1). A total of 487,689 contigs > 1 kb recovered form clean (126,119) and contaminated (358,573) soils were run through VirSorter (v 1.0.5) [72] to identify viral contigs. As described in previous protocol by Paez-Espino et al. [46], viral contigs were divided in categories 1, 2, 4, and 5 using VirSorter (v 1.0.5) and included for viral annotation, leaving 19,855 contigs from clean (5,550) and contaminated (14,305) soils, which were taken forward to for next step of the analysis as follows. Briefly, viral contigs with length >5 kb were further processed by vHMMs pipeline using 3 distinct filters criteria: 1) viral contigs had at least 5 hits to viral protein families, while the total number of genes covered by KEGG Orthology (KO) [31] of the contig was <20%; and the total number of genes covered with Pfams (v 31.0) [73]  ≤40%; 2) the number of viral protein families on the contig were equal or higher than the number of Pfams; 3) the number of viral protein families was equal or higher than 60% of the total genes. Viral contigs longer than 5 kb that met at least one of the three filtering criteria listed above were filtered out. Finally, A total of 19,292 viral contigs from clean (5387) and contaminated (13,905) soils were included for further analysis.

### Viral protein clustering and distribution

All 19,292 contigs (>1 kb) with 95% identity and 80% coverage were clustered into 18,458 viral populations (vOTUs) using ClusterGenomes (v 1.1.3), and 4,572 vOTUs larger than 10 kb were used for protein clustering using vConTACT (v 2.0) equipped with NCBI bacterial and archaeal viral RefSeq v85 database using default parameters [74]. Briefly, all-to-all protein sequence alignments were performed with DIAMOND 0.9.10 [75] to group proteins into clusters (default parameters, cut-offs of 10^−5^ on E-value and 50 on bit score). Similarity scores were determined based on the number of shared protein clusters between contigs. Contigs with bit scores >1 were processed for further clustering. After formation of the Markov algorithm clustering protein ensemble group, the viral clusters (VCs) were defined using ClusterONE (CL1) and overlapping VCs in the network were subdivided using distance-based hierarchical clustering. As a result, 4,572 vOTUs observed in the soil were divided into 909 viral clusters (Supplementary Table 4- “Contigs”, “Viral taxonomy” and “network_data_1”). The network visualization and analysis were conducted using the “Network Analysis” function in Cytoscape3.7.1 [76] (http://cytoscape.org; Supplementary Table 4- “network_data_1_parameters”). We also analyzed viral community diversity and composition using vOTU approach [77]. Briefly, 19,292 detected contigs were grouped into vOTUs (>1 kb, with greater than 95% identity and 80% coverage, based on perl script “ClusterGenomes” from https://bitbucket.org/MAVERICLab/stampede-clustergenomes/src/master/) and used for analyzing the alpha and beta diversity of viral communities. Viral taxonomy annotations were assigned using vConTACT (v 2.0) (>10 kb) by applying a majority-rules approach as previously described [78], where a viral population was adopted into a viral family if >50% of the proteins were assigned to the family with a Viral RefSeq Virus database using a BLASTp bitscore ≥50 (Supplementary Table 4- “Viral taxonomy”). Total of 273 of 4,572 vOTUs with length greater than 10 kb were successfully annotated and the rest of the vOTUs (15,781 of 18,458 vOTUs) were assigned through majority-rules approach. In total, 86% of vOTUs (15,841 of 18,548) could be assigned taxonomically at family level (Supplementary Fig. 3c).

### Virus-host linkage analysis

Three methods were used to analyze putative virus-host linkage (Supplementary Table 5): 1) Trna sequences were recovered from viromes, and aligned against all genomes in our soil metagenomes with ARAGORN (v 1.2.38) using BLAST (100% coverage and 100% sequence identity) after deleting self-hits and duplicates [46]; 2) CRISPR spacer and repeat elements were recovered from bacterial metagenomic PE reads with CRASS (v 1.2.1) [79]. According to the comparison results with viral contigs via BLASTn (100% nucleotide identity, mismatch ≤1 and E-value ≤ 10^−5^), the target spacer sequence was selected, then the repeat sequence from the same region was compared with contigs from bacterial genomes via BLASTn (E-value threshold of 10^−10^ and 100% nucleotide identity) [20]. As the two approaches obtain reliable but limited virus-bacteria relations, the third approach was used to reflect more broad conditions, by submitting viral sequences to JGI Viral Sequence Database (https://img.jgi.doe.gov/cgi-bin/vr/main.cgi) to match similar viral and putative host bacterial genomes via BLASTn (E-value threshold of 10^−5^, ≥95% sequence identity) [46].

### Bacterial and viral gene annotation

Non-redundant proteins of bacterial and viral genomes were annotated using KEGG (kobas3.0.3) [31] and CAZyme (cazydb.07312018.fa) [32] databases (Supplementary Tables 3 and 7), and viral proteins annotated by KEGG (kobas3.0.3) and Pfam (v 31.0) [73] were also used for viral genome identification and annotation (see Virus identification). Functional bacterial genes linked to carbon, nitrogen and sulfur metabolism and pesticide degradation were identified according to the metabolic pathways mapped by viral KEGG orthologs. Phosphorus metabolic genes were identified according to the utilization of phosphorus (included pathways: organic phosphorus mineralization, inorganic phosphorus hydrolysis, inorganic phosphorus solubilization, and inorganic phosphorus synthesis). Co-occurrence networks of viral and bacterial genes were visualized in Gephi (v 0.8.2) [80] based on Pearson correlation coefficients.

The viral origin of the pesticide degradation genes was validated by analyzing respective contigs in more detail VIRSorter2 (v 2.2.3, default parameters) [81], CheckV (v 0.8.1, default parameters) [36], VIBRANT (v 1.2.0, default parameters, t virome = true) [37] and DRAM-v (v 1.2.0) [38] was used to validate annotations of pesticide degradation genes. For DRAM-v, default parameters was used for AMG identification and obtained AMG flag was assigned as follows [38]: V - viral, M - metabolism flag, K - known AMG, E - experimentally verified AMG, A - viral host attachment and entry, P - peptidases for viral use, F - near the end of the contig and B – a set of consecutive genes (≥3) with metabolism flag “M”. Above data of pesticide degradation genes was shown in Supplementary Table 9-“Gene information” and “DRAM-v information”. Sigma-70 transcriptional promoter was recognized by SAPPHIRE (*p* < 0.05, https://sapphire.biw.kuleuven.be/index.php) [82], FindTerm (energy threshold value < −12.0, http://www.softberry.com/berry.phtml?topic=findterm&group=programs&subgroup=gfindb) [83] and RhoTermPredict (RUT site C/G ratio>1 with regularly spaced cytosine residues within the window (every 11–13 nt), and palindromic score >6) [84] were used to predict the Rho-independent and Rho-dependent terminators, respectively. For phylogenetic analysis, the top 20 most similar protein sequences from NCBI RefSeq database and 9 protein sequences from bacterial dataset to our viral L-2-haloacid dehalogenase gene were retrieved using BLASTp. Protein sequences from local bacterial dataset were selected with a threshold of identity ≥ 40%, coverage ≥40% and E-value < 10^−5^. After alignment with ClusterW, MEGA 7 [67] was used to construct a maximum likelihood tree (*n* = 500 bootstraps), and visualized in iTOL. Protein models of pesticide degradation genes were constructed using Phyre2 [41], and viral contig maps were constructed using Easyfig (v 2.2.4) [85].

To explore the variance in viral gene functions in association with pesticide stress, predicted viral proteins annotated by Virus Orthologous Groups database (VOGDB, vog203, http://vogdb.org/) and Pfam database for screening out core functional genes (COREs) and auxiliary metabolic genes (AMGs) were used, respectively (Supplementary Table 8). Genes linked to viral replication (Xr) and viral structure (Xs), such as capsid, integrase, and holin associated genes, were defined as “core functional genes”, which would be more concerned with the proliferation process of the virus itself than with hallmark genes. Hallmark genes also include genes commonly identified as the viral source while functions are hard to classify or functions unknown by VOGDB. Proteins involved in nutrient transformation and pollutant degradation were defined as auxiliary metabolic genes [50, 86, 87]. Finally, a total of 28,686 core functional genes from 261 groups and consisted of 3,310 AMGs belonging to 229 Pfam families were used for downstream analysis. A log10 transformation was used to better visualize the relative abundances of viral core functional genes and AMGs in Supplementary Fig. 7a.

### L-DEX Gene synthesis and protein expression validation

To confirm functioning of virus-encoded AMGs for pesticide degradation we chose one commonly observed candidate gene: the gene encoding L-2-haloacid dehalogenase (L-DEX, EC:3.8.1.2) from CON_VIRSorter_k127_2354611, which is involved in degradation of L-2-haloacids. The gene was synthesized by PCR-based accurate synthesis (PAS) and then cloned into pET-32a (+) plasmid, which was transferred into *E. coli* TOP10 strain. The positive clones were screened by LB agar plates with 50 μg mL^−1^ Ampicillin and the target gene was verified by PCR sequencing. The recombinant plasmid pET-32a (+)-LDEX was transformed into *E. coli* ArcticExpress (DE3) and the protein expression was induced with 0.5 mM IPTG at 37 °C for 4 h. After cell lysis by sonication (400 W, with each 4 s being interrupted by 8 s, total 20 min) and centrifugation, the target protein L-2-haloacid dehalogenase existed in the form of inclusion body. After the solubilization of the purified inclusion bodies, the target protein fraction was purified using Ni-IDA affinity column (Novagen) and examined by 12% SDS–PAGE. The qualitative and quantitative protein expression was determined by Western Blot.

### Protein activity verification

The activity of purified virus-encoded L-2-haloacid dehalogenase was determined by measuring the amount of Cl^−^ that was produced after monochloroacetate (MCA, CAS: 79-11-8) and S(L)-2-chloropropionic acid (S(L)-2-CPA, CAS: 29617-66-1) reaction as follows. Briefly, 1 M MCA or S-2-CPA was added to 1 mL Glycine-NaOH Buffer (100 mM, pH = 10.0) and configured into a reaction system with a final concentration of 10 mM. Then 10 μg virus-encoded L-2-haloacid dehalogenase (0.5 μg mL^−1^) was added, and the reaction ran at 37 °C for 5 h and 24 h followed by addition of 10 μL H_3_PO_4_ (85%, *w/w*) to terminate the reaction (four replicates per treatment). Treatments without enzyme, and with inactivated enzyme (enzyme inactivated at 99 °C for 10 min) were used as negative controls. Spectrophotometric method of mercury sulfocyanide was used to determine the Cl^-^ generated in the reaction at 480 nm using EnSight™ Multimode Microplate Reader (PerkinElmer, Singapore), and the residual amount of the substrate in the reaction was determined indirectly.

The toxic effect of MCA and S-2-CPA on bacteria was determined in the absence and presence of phage-encoded L-DEX. The minimum inhibitory concentration (MIC) of two substrates against the donor strain *E. coli* ArcticExpress without L-DEX gene (named “*E. coli”*), *E. coli* ArcticExpress with L-DEX plasmid (named “*E. coli* + *L-DEX”*), *E. coli* ArcticExpress with L-DEX plasmid induced by 0.4 mM IPTG (named “*E. coli* + *L-DEX* + *IPTG*”) were first determined. Specifically, LB liquid medium with a final concentration of 6-10 mM MCA and 9-13 mM S-2-CPA were mixed with bacterial broth (OD 0.6-0.8) as a ratio of 100:1 and incubated at 37 °C for 12 h. The bacterial growth was recorded as turbidity at 600 nm (OD_600_) using UV spectrophotometer (LabTech UV8100B, China) and minimum concentration (MIC) determined as complete inhibition of bacterial growth. Based on these results, bacterial growth curves were quantified also at sub-MIC MCA (7 mM) and S-2-CPA (10 mM) concentrations for 24 h at 37 °C, respectively.

### Data statistical analysis

Data statistics and visualization in this study were performed using GraphPad Prism 8.0 (https://www.graphpad.com/) and R (v 3.6.2) (https://www.r-project.org/). The microbial rarefaction curve and alpha and beta diversity analyses (including alpha index, UPGMA and NMDS), were conducted using vegan and ggplot2 packages in R. The interpretation degree (*R* value) and significance (*p* value) between the samples were calculated based on Adonis analysis. For example, Adonis *R*^*2*^ = 0.99 indicates that grouping based on the contamination vs. no contamination explained a 99% of between sample variance and *p* < 0.05 value indicates high statistical significance. Stress value <0.05 in NMDS based on Bray–Curtis distance indicates that the NMDS analysis results have good conformity and that the distance between samples in reduced 2-dimensional space corresponds with the actual multivariate distance between the samples. Unweighted pair group method with arithmetic mean (UPGMA) is also used to cluster the samples, which is a simple hierarchical clustering method based on pairwise similarity matrix (or a dissimilarity matrix). Pearson correlation between genes was calculated using psych package, leaving the correlation with a threshold of |*r*| > 0.6 and *p* < 0.05 to generate the network via Gephi (v. 0.8.2) [80]. LEfSe analysis was performed using online platform Galaxy (https://huttenhower.sph.harvard.edu/galaxy/). A combination of ANOVA, Tukey’s multiple comparisons test, *T*-tests, and non-parametric Fisher’s exact and Wilcoxon rank sum tests were used for the statistical analysis using Graphpad Prism 8.0.

## Supplementary information

Supplementary information

Supplementary Table 3

Supplementary Table 4

Supplementary Table 5

Supplementary Table 7

Supplementary Table 8

Supplementary Table 9

## Data Availability

The bacterial and viral raw metagenome sequence data generated in this study are archived at Genome Sequence Archive (Genomics, Proteomics & Bioinformatics 2017, https://bigd.big.ac.cn/gsa) and National Genomics Data Center [88], Beijing Institute of Genomics (China National Center for Bioinformation), Chinese Academy of Sciences, under accession number PRJCA003886. In addition, 19,292 viral contigs have been deposited in the Genome Warehouse (https://bigd.big.ac.cn/gwh) under accession numbers GWHBCHI00000000. All data are publicly accessible and can be download from https://ngdc.cncb.ac.cn/bioproject/browse/PRJCA003886.
