# Supplementary material for: Organochlorine contamination enriches virus-encoded metabolism and pesticide degradation associated auxiliary genes in soil microbiomes
Source: ISME J. 2022 Jan 17;16(5):1397–408. doi: 10.1038/s41396-022-01188-w (PMC9038774; doi:10.1038/s41396-022-01188-w)
Supplement: Supplementary file 1 — Supplementary information [file 41396_2022_1188_MOESM1_ESM.docx]

**Supplementary information**

**Supplementary Table 1.** Assembly summary for bacterial and viral metagenomes.

| **File** | **Clean virome metagenomes** | | | **OCP-contaminated virome metagenomes** | | | | | |
| --- | --- | --- | --- | --- | --- | --- | --- | --- | --- |
|  | C1**-**C3 | | | S1**-**S6 | | | | | |
| Min sequence length | 200 | | | 200 | | | | | |
| Max sequence length | 326943 | | | 592406 | | | | | |
| Total sequence number | 1091906 | | | 3786311 | | | | | |
| N20 | 3953 | | | 3290 | | | | | |
| N50 | 822 | | | 687 | | | | | |
| N90 | 336 | | | 328 | | | | | |
| Total sequence length | 780285451 | | | 2532521380 | | | | | |
| GC Number | 482387313 | | | 1471313891 | | | | | |
| GC content | 0.618219 | | | 0.580968 | | | | | |
| Sequences greater than 1kb | 129116 | | | 358573 | | | | | |
| **File** | **Clean bacterial metagenomes** | | | **OCP-contaminated bacterial metagenomes** | | | | | |
|  | C1 | C2 | C3 | S1 | S2 | S3 | S4 | S5 | S6 |
| Min sequence length | 200 | 200 | 200 | 200 | 200 | 200 | 200 | 200 | 200 |
| Max sequence length | 78630 | 77436 | 61785 | 173252 | 104301 | 1211824 | 84598 | 78516 | 349174 |
| Total sequence number | 2259556 | 2413072 | 2421002 | 5895330 | 5942138 | 6480899 | 6348301 | 6313873 | 5710577 |
| N20 | 903 | 875 | 1019 | 906 | 1008 | 1001 | 1002 | 982 | 913 |
| N50 | 511 | 502 | 539 | 515 | 544 | 545 | 542 | 539 | 518 |
| N90 | 328 | 330 | 334 | 319 | 328 | 333 | 332 | 332 | 317 |
| Total sequence length | 1174987327 | 1242046504 | 1315714334 | 3090674583 | 3242382906 | 3544760466 | 3462076323 | 3422849767 | 3001654266 |
| GC Number | 733070959 | 770559945 | 823596339 | 2049203432 | 2147229717 | 2361614709 | 2305121808 | 2263579744 | 1991334444 |
| GC content | 0.623897 | 0.620395 | 0.625969 | 0.663028 | 0.662238 | 0.666227 | 0.665821 | 0.661314 | 0.663412 |
| Sequences greater than 1kb | 128949 | 123769 | 161924 | 320892 | 392775 | 426228 | 417591 | 404346 | 319667 |

**Supplementary Table 2.** Physico-chemical properties and pesticide content in clean (C1-C3) and OCP-contaminated soils (Light contamination: S1-S3; Heavy contamination: S4-S6). The United States Environmental Protection Agency (EPA) screening Levels of residential soil or industrial soil corresponding to compounds, as well as the CAS number, are shown in the last three columns

|  | **Indicators** |  | **C1** | **C2** | **C3** | **S1** | **S2** | **S3** | **S4** | **S5** | **S6** | **Resident Soil (EPA)** | **Industrial Soil (EPA)** | | **CAS** |
| --- | --- | --- | --- | --- | --- | --- | --- | --- | --- | --- | --- | --- | --- | --- | --- |
| **Physico-chemical properties** | **pH** |  | 5.7 ± 0.1 b | 5.8 ± 0.1 b | 6.0 ± 0.1 a | 7.6 ± 0.1 b | 7.6 ± 0.3 a | 7.9 ± 0.1 a | 7.9 ± 0.1 a | 7.9 ± 0.2 a | 7.9 ± 0.1 a |  |  |  | |
|  | **Cation Exchange Capacity** | **cmol·kg^-1^** | 17.4 ± 0.3 b | 17.3 ± 1.0 b | 18.5 ± 0.9 a | 23.4 ± 0.3 a | 22.6 ± 1.0 a | 22.3 ± 0.9 a | 21.4 ±1.3 a | 21.9 ± 2.4 a | 19.8 ± 1.15 a |  |  |  | |
|  | **Soil Organic Matter** | **g·kg^-1^** | 46.0 ± 0.4 b | 49.2 ± 0.5 a | 49.5 ± 0.5 a | 61.2 ± 0.6 a | 52.6± 1.0 b | 49.1 ± 0.4 c | 46.4 ±1.1 c | 38.9 ± 2.1 d | 38.1 ± 0.9 d |  |  |  | |
|  | **Total Nitrogen** | **g·kg^-1^** | 1.4 ± 0.1 a | 1.4 ± 0.3 b | 1.4 ± 0.3 c | 3.3 ± 0.1 a | 3.1 ± 0.3 b | 2.7 ± 0.3 c | 2.7 ± 0.1 c | 2.2 ± 0.1 d | 2.2 ± 0.2 d |  |  |  | |
|  | **Total Phosphorus** | **g·kg^-1^** | 0.9 ± 0.1 a | 0.8 ± 0.1 a | 0.8 ± 0.1 a | 3.4 ± 0.1 a | 3.2 ± 0.2 ab | 2.9 ± 0.1 bc | 2.5 ± 0.3 d | 2.6 ± 0.2 cd | 1.7 ± 0.1 e |  |  |  | |
|  | **Available Sulfur** | **mg·kg^-1^** | 27.6 ± 1.3 b | 28.8 ± 0.3 ab | 30.6 ± 0.6 a | 45.9 ± 0.3 a | 37.9 ± 0.6 b | 29.9 ± 0.1 d | 33.4 ±0.1 c | 27.8 ± 0.2 e | 29.9 ± 0.7 d |  |  |  | |
| **Pesticide content** | ***ortho-* and *para-*Nitrochlorobenzene** | **mg·kg^-1^** | ND | ND | ND | 3.6 ± 0.1 d | **24.2 ±1.4 c** | 9.1 ± 0.9 d | **30.8 ± 1.1 c** | **253.2 ± 5.6 a** | **115.2 ± 15.2 b** | 10.80 | 45.7 | 88-73-3; 100-00-5 | |
|  | ***meta-*Nitrochlorobenzene** | **mg·kg^-1^** | ND | ND | ND | 5.7 ± 0.3 d | 14.5 ± 0.6 b | 10.8 ± 1.6 c | 14.4 ± 0.4 b | 14.3 ± 0.7 b | 17.3 ± 3.2 a |  |  |  | |
|  | **Benzene** | **mg·kg^-1^** | ND | ND | ND | **2.1 ± 0.4 d** | **7.8 ± 0.2 b** | **4.3 ± 0.3 cd** | **7.9 ± 0.2 b** | **5.7 ± 0.7 bc** | **64.4 ± 2.3 a** | 1.20 | 5.1 | 71-43-2 | |
|  | **Chlorobenzene** | **mg·kg^-1^** | ND | ND | ND | 84.5 ± 4.1 e | 95.1 ±7.3 e | 119.4 ± 4.4 d | 197.1 ± 4.1 c | **330.2 ± 8.1 a** | **289.1 ± 19.6 b** | 280.00 | 1300 | 108-90-7 | |
|  | **Ethylbenzene** | **mg·kg^-1^** | ND | ND | ND | **21.0 ± 1.5 f** | **43.9 ±0.4 e** | **30.7 ± 2.1 d** | **50.3 ± 3.3 c** | **81.9 ± 5.2 b** | **175.4 ± 22.0 a** | 5.80 | 25 | 100-41-4 | |
|  | ***para-*Dichlorobenzene** | **mg·kg^-1^** | ND | ND | ND | **4.1 ± 0.2 e** | **6.1± 0.9 d** | **4.8 ± 0.3 e** | **28.2 ± 1.4 b** | **20.8 ± 0.5 c** | **41.2 ± 2.5 a** | 2.60 | 11 | 106-46-7 | |
|  | ***meta-*Dichlorobenzene** | **mg·kg^-1^** | ND | ND | ND | 15.3 ± 0.5 e | 44.1 ±2.2 b | 23.3 ±0.9 d | 21.6 ±1.1 de | 30.9 ± 1.6 c | 132.7 ± 14.4 a |  |  | 541-73-1 | |
|  | ***ortho*-Dichlorobenzene** | **mg·kg^-1^** | ND | ND | ND | 145.1 ± 18.8 e | 202.2 ±7.3 b | 307.4 ± 27.9 d | 733.4 ± 42.7 de | 1440.2 ± 233.5 c | **3760.5 ± 344.6 a** | 1800.00 | 9300 | 95-50-1 | |
|  | **Total** | **mg·kg^-1^** | ND | ND | ND | 281.3 ± 21.4 e | 437.9 ± 8.5 d | 509.8 ± 28.7 d | 1083.7 ±40.4 c | 2177.2 ± 241.5 b | 4595.8 ± 344.0 a |  |  |  | |

Note: Values are shown as means ± standard deviation of triplicate measurements based on Tukey's multiple comparisons test. ND: not detected. Concentrations higher than resident soil screening values of EPA are bolded and values higher than industrial soil screening baseline are underlined.

**Supplementary Table 3.** Bacterial taxonomy (named “Taxonomy”) and relative abundance of annotated genes based on KEGG orthologs (named “KEGG annotation of bacteria”) and CAZy subfamilies (named “CAZy annotation of bacteria”) in clean (C1-C3) and OCP-contaminated soils (Light contamination: S1-S3; Heavy contamination: S4-S6).

**Supplementary Table 4.** Overview of the viral contig relative abundances, viral populations (vOTUs), viral clustering results (VCs) and network data. Sheet 1: Viral contig information, including TPM values, relative abundances, viral operational taxonomic unit (vOTUs), viral clusters (VCs) and presence in clean (C1-C3) and OCP-contaminated soils (Light contamination: S1-S3; Heavy contamination: S4-S6), named “Contigs”. Sheet 2: Virus taxonomy and method information (VCONTACT2 or Majority-rules approach), named “Virus taxonomy”. Sheet 3: Gene-sharing network result in clean and OCP-contaminated soils (output from VConTACT 2.0; named “network_data_1”). Sheet 4: Parameters of network_data_1 exported from Cytoscape 3.7.1 (named “Network_data_1_parameters”).

**Supplementary Table 5.** Information on predicted virus-host linkages. Table shows predicted host taxa information corresponding to viral contigs and methods for obtaining these associations.

**Supplementary Table 6.** Average number of genes linked to nutrient transformation and pesticide degradation in annotated bacterial and viral metagenomes derived from clean and OCP-contaminated soils.

| Types | Bacterial genes | | | Viral genes | | |
| --- | --- | --- | --- | --- | --- | --- |
|  | Clean | Light | Heavy | Clean | Light | Heavy |
| C (carbon) | 584 | 589 | 586 | 19 | 27 | 27 |
| N (nitrogen) | 48 | 48 | 49 | 2 | 3 | 3 |
| P (phosphorus) | 7 | 8 | 7 | 1 | 1 | 1 |
| S (sulphur) | 69 | 70 | 69 | 0 | 2 | 2 |
| Pesticides | 121 | 129 | 126 | 0 | 2 | 2 |
| Genes included in two functions | 28 | 37 | 37 | 0 | 0 | 0 |

**Supplementary Table 7.** Annotated viral genes based on KEGG and CAZy databases. The first two sheets show KEGG orthologs (named “KEGG annotation of virus”) and CAZy subfamilies (named “CAZy annotation of virus”) of viral genes, as well as TPM values and relative abundances in clean (C1-C3) and OCP-contaminated soils (Light contamination: S1-S3; Heavy contamination: S4-S6). The third sheet shows selected functional genes linked to carbon (C), nitrogen (N), phosphorus (P), sulfur (S) cycling and pesticide degradation selected based on KEGG annotation dataset (named “Summary of selected genes”).

**Supplementary Table 8.** Viral Pfam families (named “Pfam families”), core functional gene (named “Core functional genes”) based on VOGDB and AMG (named “AMGs”) abundances based on Pfams. The annotation references for Pfam function classifications are displayed with DOI.

**Supplementary Table 9.** Detailed information on viral pesticide degradation genes, including promoter and terminator predicted region, VIRSorter2, checkV, VIBRANT (named “Gene information”) and DRAM-v results (named “DRAM-v information”) and similar protein sequences to L-DEX selected from NCBI RefSeq database and local bacterial dataset (named “L-DEX BLASTp query”).


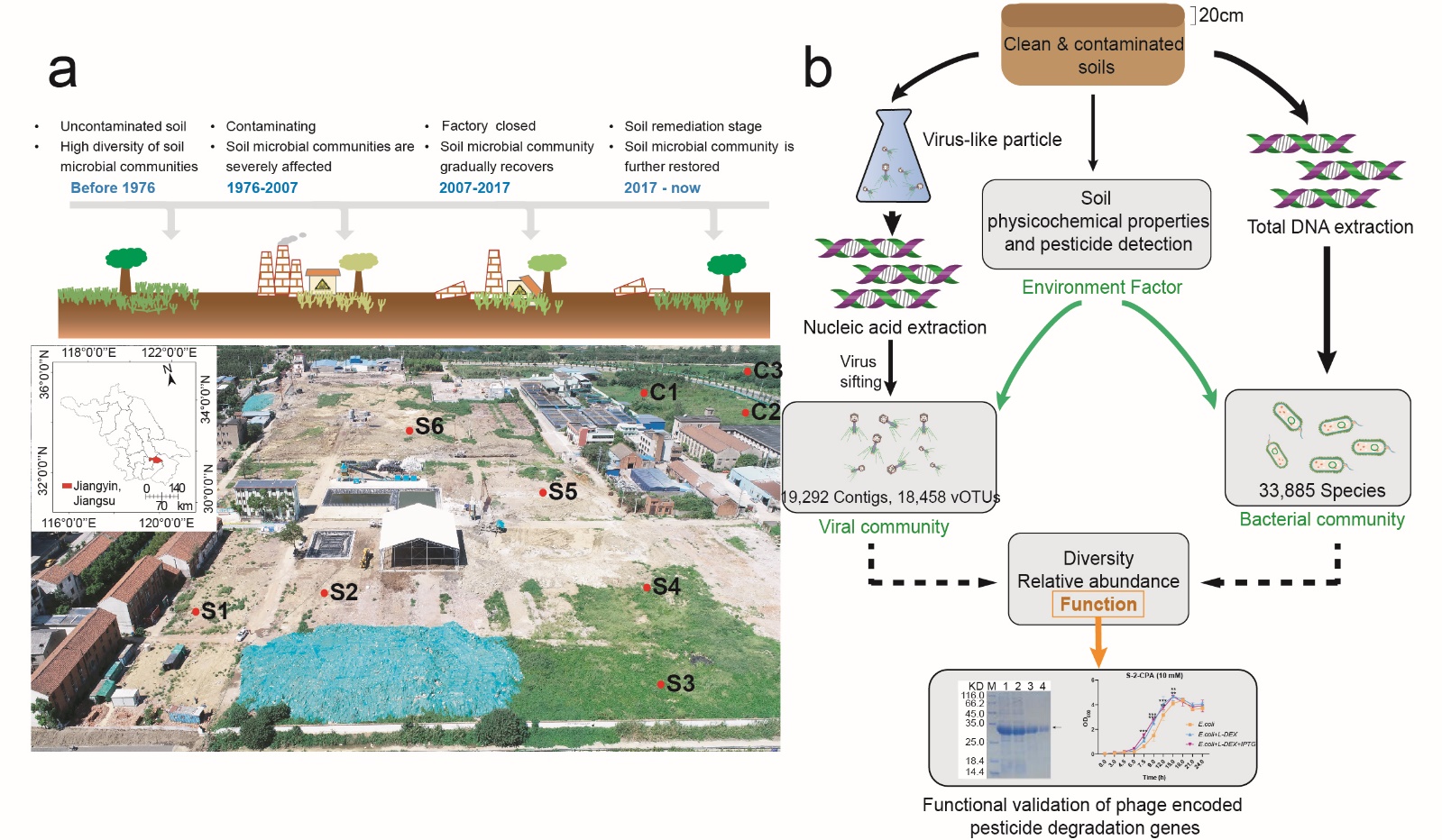


**Supplementary Fig. 1.** Sampling sites of clean (C1-C3) and OCP-contaminated (Light contamination: S1-S3; Heavy contamination: S4-S6) soil samples **(a)** and a conceptual figure **(b)** describing the experimental methods and approaches used in this study.

**
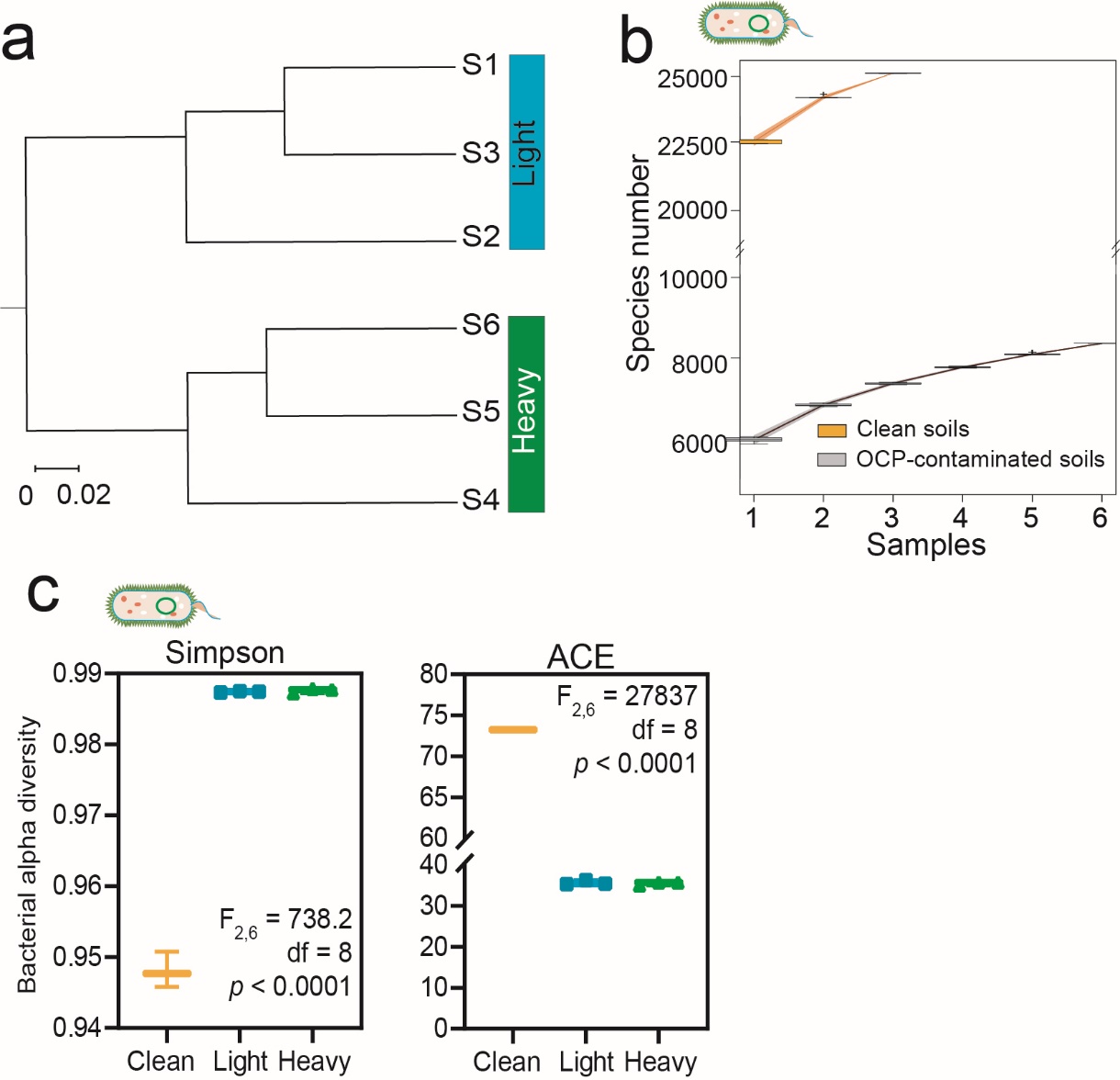
**

**Supplementary Fig. 2. a,** OCP-contaminated soils classified into light and heavy contamination levels according to UPGMA clustering based on Bray-Curtis similarity matrix of pesticide contents. **b,** Bacterial species accumulation analysis in clean (C1-C3) and OCP-contaminated soils (Light contamination: S1-S3; Heavy contamination: S4-S6)**. c,** Differences in Simpson and ACE index of bacterial communities between clean (C1-C3) and OCP-contaminated soils (Light contamination: S1-S3; Heavy contamination: S4-S6). ANOVA followed by Tukey’s multiple comparisons test was used to compare differences between groups.


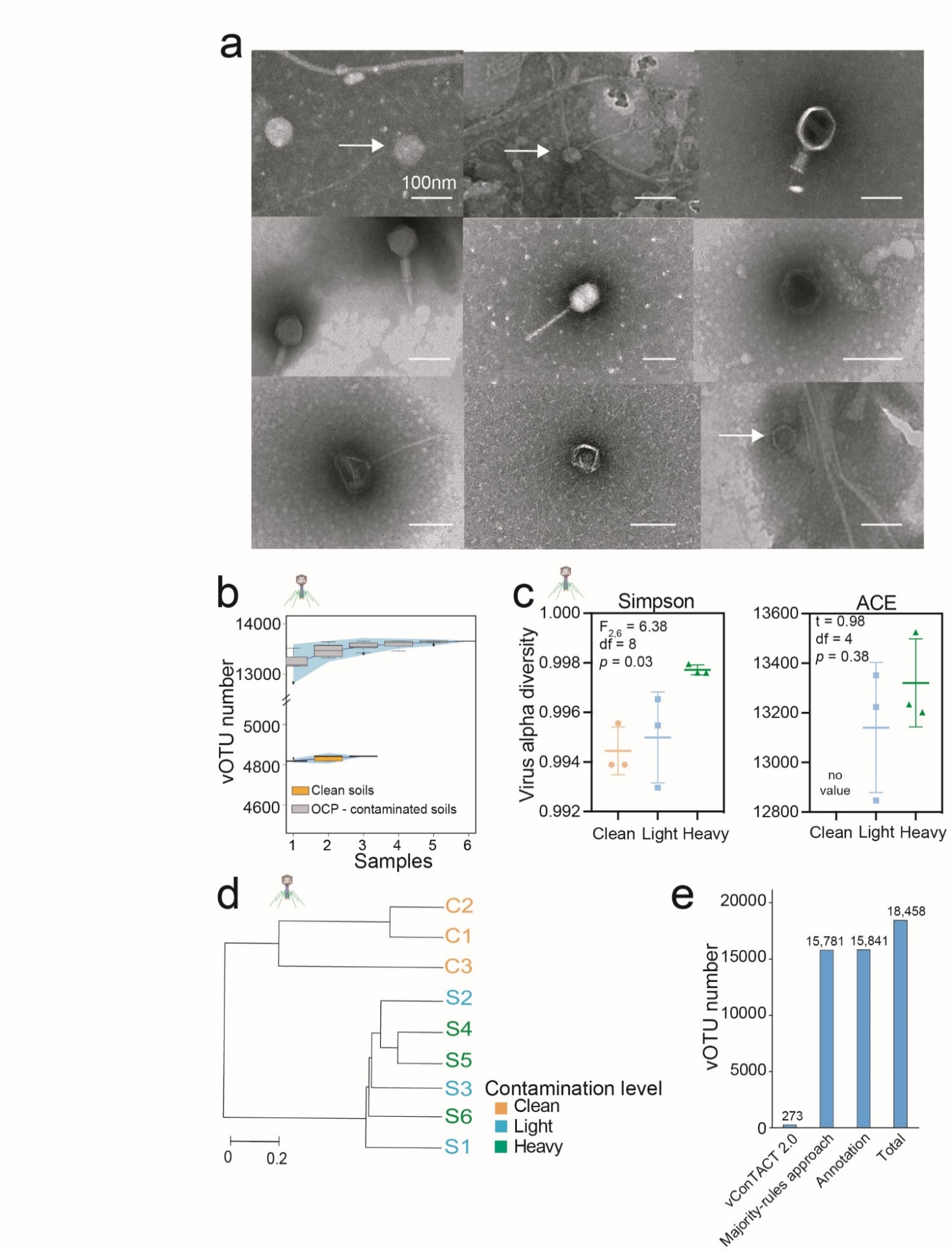


**Supplementary Fig. 3 a,** Representative virus morphotypes observed in clean and OCP-contaminated soils based on transmission electron microscopy (Scale bar: 100 nm). **b,** vOTU accumulation analysis in clean (C1-C3) and OCP-contaminated soils (Light contamination: S1-S3; Heavy contamination: S4-S6)**. c,** Differences in Simpson and ACE index of viral communities between clean (C1-C3) and OCP-contaminated soils (Light contamination: S1-S3; Heavy contamination: S4-S6). **d,** Classification of soil viromes based on viral clusters (VCs) and UPGMA clustering analysis. **e,** The number of viral family taxonomy annotations based on two methods. ANOVA followed by Tukey’s multiple comparisons test was used to compare differences between groups.

**
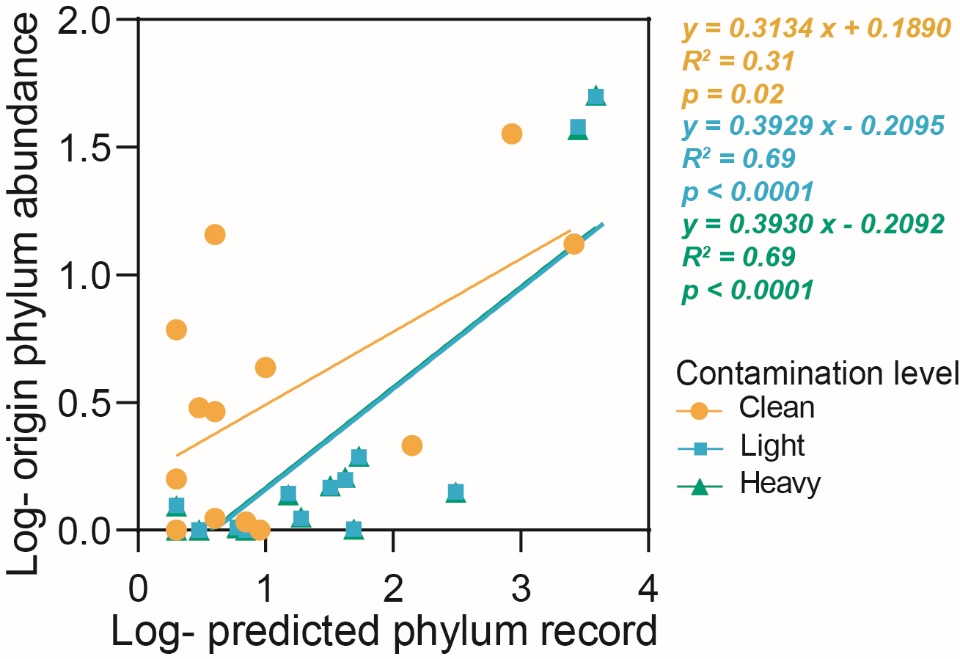
**

**Supplementary Fig. 4** Linkage-specific host number-abundance pattern in clean (C1-C3) and OCP-contaminated soils (Light contamination: S1-S3; Heavy contamination: S4-S6). The X- axis shows the log_2_(n+1), where n is the number of predicted host bacteria, while the Y- axis shows the log_2_(n+1), where n is the relative abundance of bacteria in metagenomes.

**
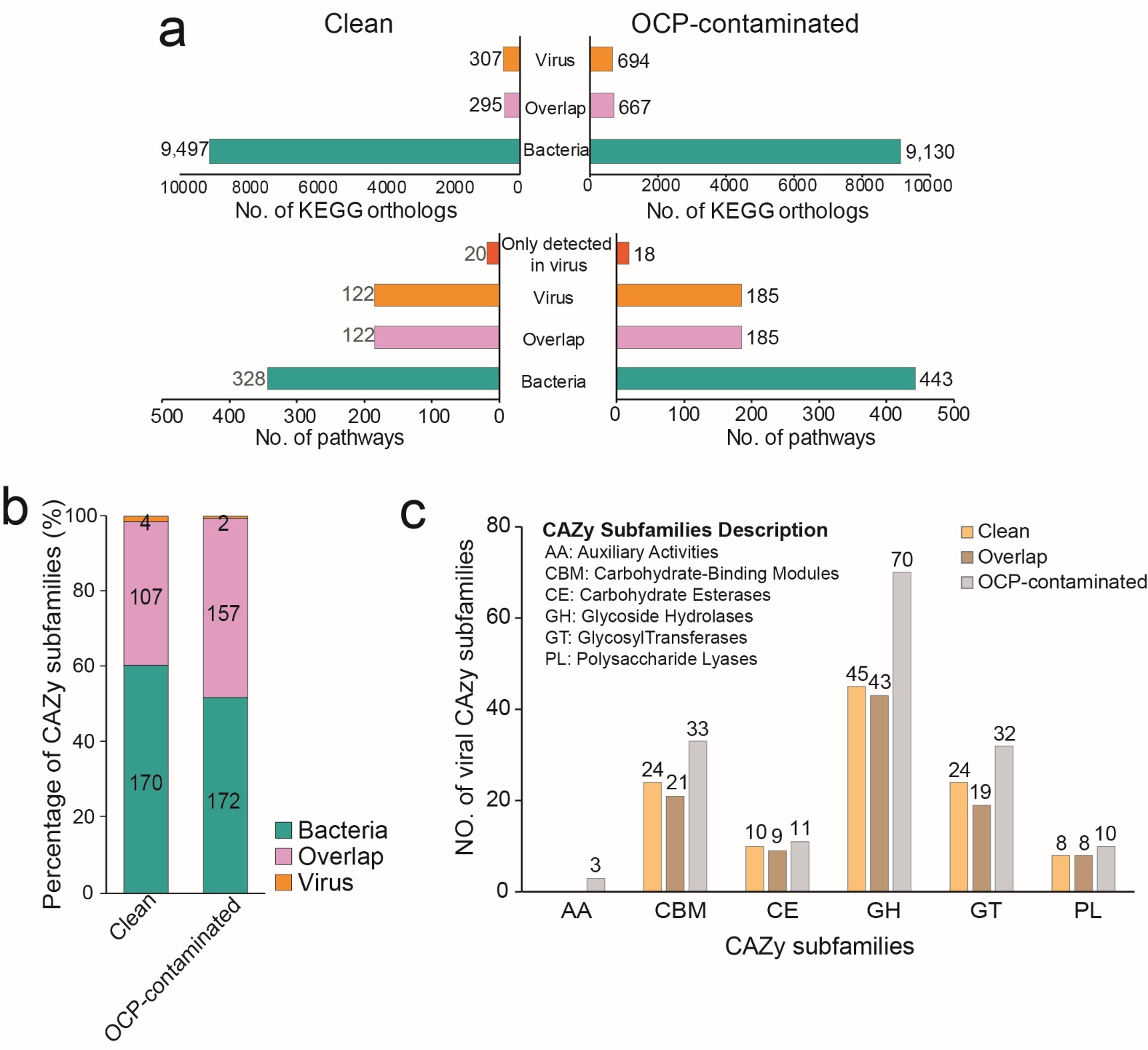
**

**Supplementary Fig. 5 a,** Total number of KEGG orthologs (KO) and KEGG pathways of bacterial and viral genomes in clean (C1-C3) and OCP-contaminated soils (Light contamination: S1-S3; Heavy contamination: S4-S6). “Only detected in virus” denotes for pathways that were exclusively detected in soil virome. **b,** Total number and percentage of CAZy subfamilies of bacterial and viral genes in clean (C1-C3) and OCP-contaminated soils (Light contamination: S1-S3; Heavy contamination: S4-S6). **c,** The average number of CAZy subfamilies observed in viral metagenomes in clean (orange) and OCP-contaminated (grey) soils (brown bar shows the overlap between both soils).

**
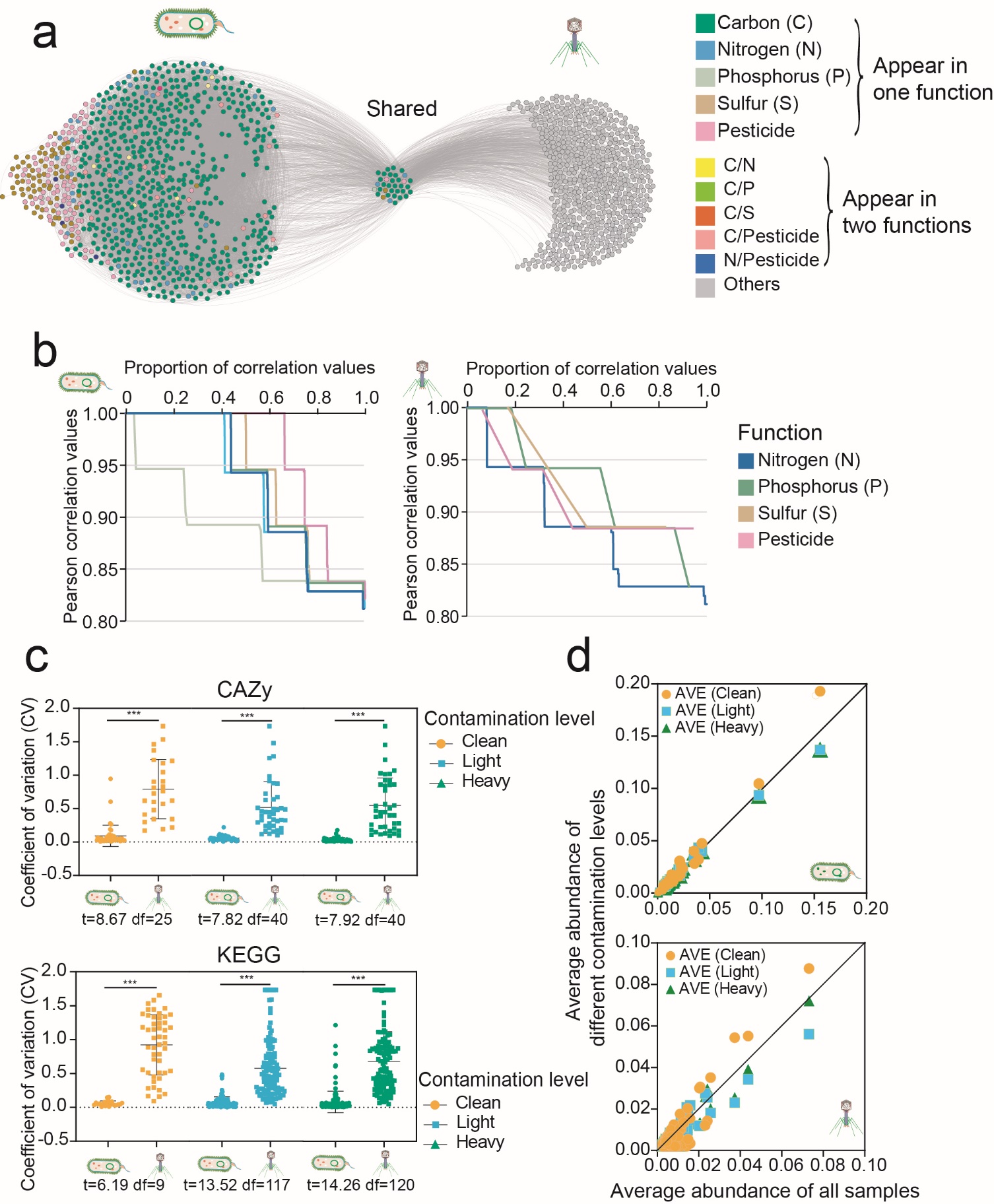
**

**Supplementary Fig. 6 a,** Co-occurrence networks of functional genes between bacteria and viruses. Lines indicate significant Pearson correlations (*p*<0.05 and |*r*|>0.6) between genes (nodes) and node colors indicate different functional genes as described in the legend. Genes linked to carbon (C), nitrogen (N), phosphorus (P), sulfur (S) metabolism and pesticide-degradation are shown in green, blue, light blue, brown and pink colors, respectively. The other colors denote genes that were associated with two functions, and “Others” on grey color the other metabolic genes we did not focus in this study. **b,** Pearson correlations (*p*<0.05 and |*r*|>0.6) between nitrogen (N - blue), phosphorus (P - grey), sulfur (S -brown) metabolism and pesticide-degradation (pink) genes with carbon (C - green) metabolism genes in soil bacterial and viral communities. **c,** The coefficient of variation of 199 AMGs (151 KEGG and 48 CAZyme) viral and bacterial genes in clean, light and heavy contaminated soil. Paired t-tests were used to compare differences between groups. **d,** Correlation between the 199 AMG abundances in all soil samples (X-axis) with average AMG abundances in clean and OCP-contaminated soil samples (orange, green and blue colors; Y-axis).

**
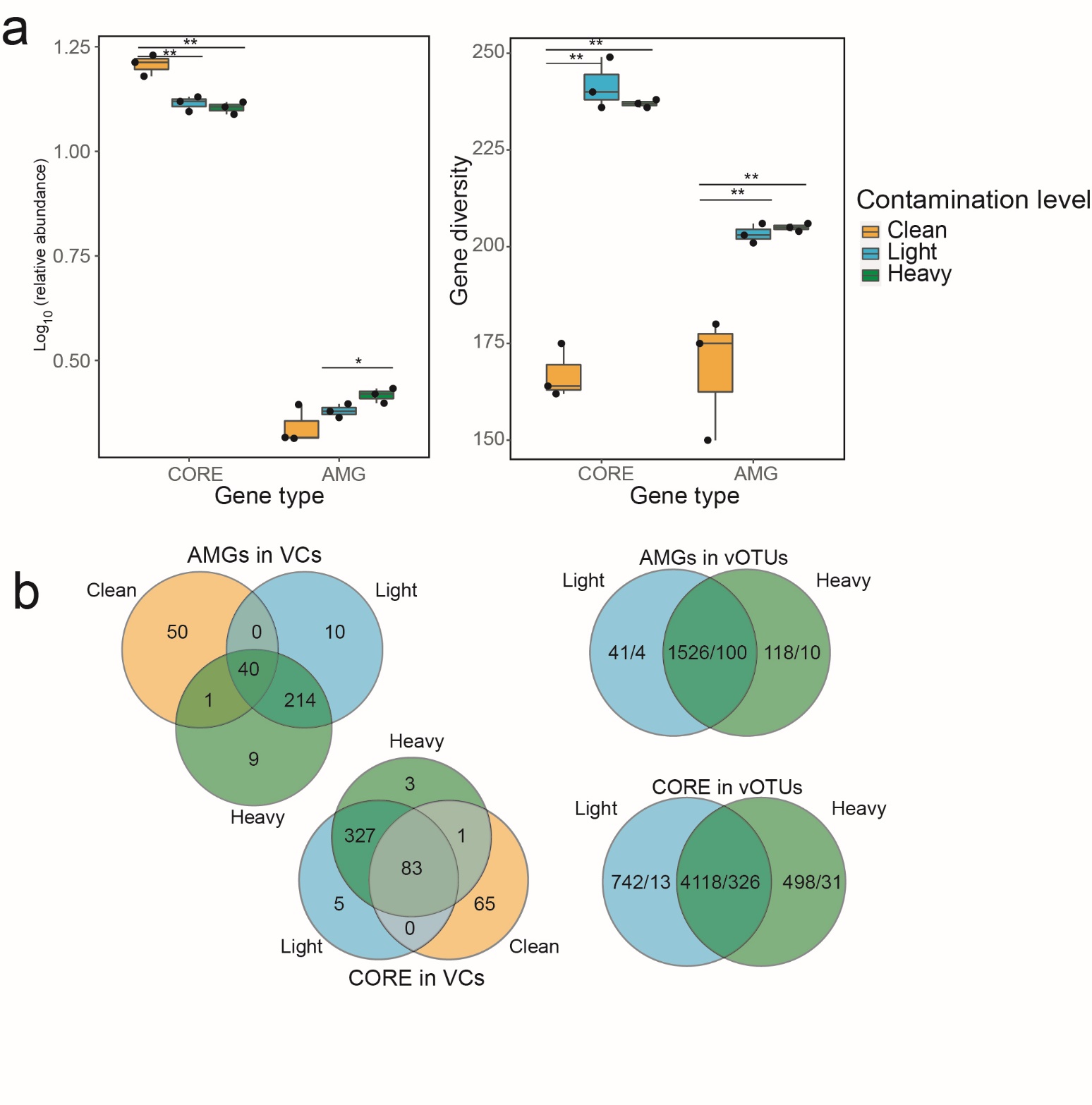
**

**Supplementary Fig. 7 a,** Log _10_ (relative abundance) of viral core functional genes (CORE) and auxiliary metabolic genes (AMG) in clean (C1-C3), light (S1-S3) and heavy (S4-S6) contaminated soils. ANOVA followed by Tukey’s multiple comparisons test was used to compare differences between groups. **b,** Venn diagrams showing the number of unique and shared core functional genes and AMGs based on VCs (left) and vOTUs (right) in clean (C1-C3; orange) and OCP-contaminated soils (Light contamination: S1-S3; blue; Heavy contamination: S4-S6; green)**.** On the right, the first and second number denotes for AMGs or core functional genes detected in vOTUs versus VCs.


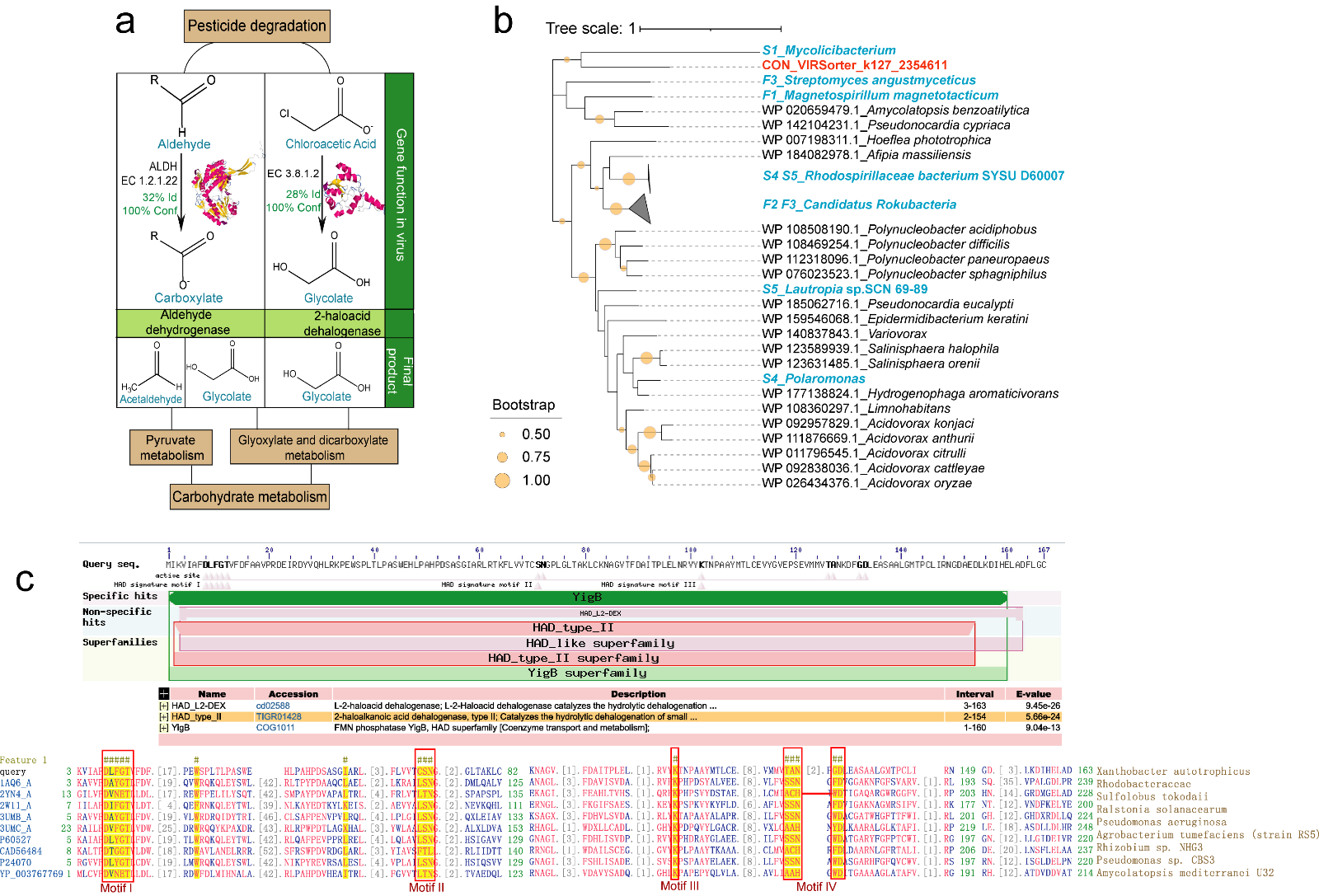


**Supplementary Fig. 8 a,** Overview of pesticide degradation genes observed in soil virome. From left to right: aldehyde dehydrogenase and 2-haloacid dehalogenase genes. Final products predicted by KEGG metabolism pathways are linked to the next KEGG pathway below. Protein structure of pesticide degradation genes were modelled using Phyre2. **b,** Phylogenetic protein tree based on L-2-haloacid dehalogenase of virus CON_VIRSorter_k127_2354611 (on red) and similar protein sequences retrieved from bacterial metagenomic dataset (named as “sample + bacteria taxa” on blue) and NCBI RefSeq database (on black) via BLASTp. The tree was bootstrapped 500 times and bootstrap estimates are shown on yellow circles. **c,** Protein conserved domains query and alignment results of virus-encoded L-2-haloacid dehalogenase. The CD-Search standard results list the highest scoring domain models in each source database. Active site is denoted by #, and signature motif site is denoted by red frame. Red and blue text colors indicate highly (Bit > 2.0) and less (Bit < 2.0) conserved domains.

**
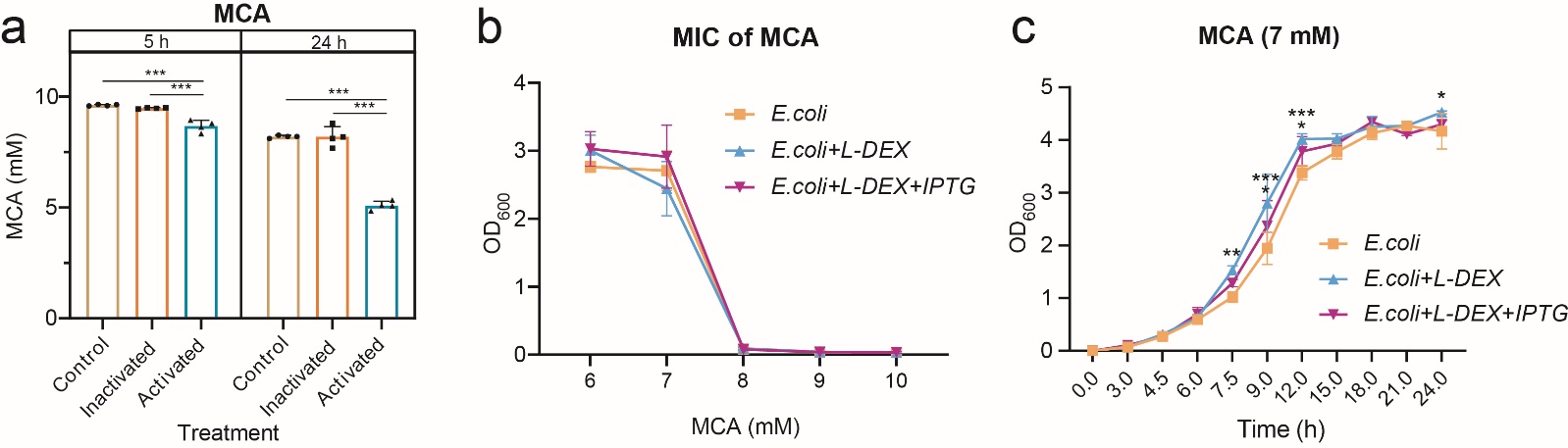
**

**Supplementary Fig. 9 a,** Enzymatic activity (MCA breakdown) of purified L-DEX in control (no protein added), inactivated (protein deactivated by high temperature) and activated (protein added) treatments, respectively. **b,** Minimum inhibitory concentration (MIC) of MCA on *E. coli*. **c,** *E. coli* growth curve under 7 mM MCA environment. *E. coli, E. coli+L-DEX* and *E. coli+L-DEX+IPTG* show the donor strain *E.* *coli* ArcticExpress without L-DEX gene, *E.* *coli* ArcticExpress with L-DEX gene and *E.* *coli* ArcticExpress with L-DEX gene induced by 0.4 mM IPTG, respectively. ANOVA followed by Tukey’s multiple comparisons test was used to compare differences between groups.
